# FGL1: a novel biomarker and target for non-small cell lung cancer, promoting tumor progression and metastasis through KDM4A/STAT3 transcription mechanism

**DOI:** 10.1186/s13046-024-03140-6

**Published:** 2024-08-01

**Authors:** Tian Yao Liu, Jin Shan Yan, Xin Li, Lu Xu, Jun Li Hao, Su Ya Zhao, Qi Lin Hu, Fang Jian Na, He Ming Li, Yue Zhao, Ming Fang Zhao

**Affiliations:** 1 Department of Medical Oncology, The First Hospital of China Medical University, No.155 Nanjingbei Road, Shenyang, Liaoning, 110001 People’s Republic of China; 2 Network Information Center, China Medical University, Shenyang, China; 3 Guangdong Association of Clinical Trials (GACT)/Chinese Thoracic Oncology Group (CTONG) and Guangdong Provincial Key Lab of Translational Medicine in Lung Cancer, Guangzhou, China; 4 Department of Cell Biology, Key Laboratory of Medical Cell Biology, Ministry of Education, School of Life Sciences, China Medical University, Shenyang City, Liaoning Province, 110122 China

**Keywords:** FGL1, KDM4A, Stat3, NSCLC, Transcriptional regulation

## Abstract

Non-small cell lung cancer (NSCLC) is characterized by a high incidence rate and poor prognosis worldwide. A deeper insight into the pathogenesis of NSCLC and identification of novel therapeutic targets are essential to improve the prognosis of NSCLC. In this study, we revealed that fibrinogen-like protein 1 (FGL1) promotes proliferation, migration, and invasion of NSCLC cells. Mechanistically, we found that Stat3 acts as a transcription factor and can be recruited to the FGL1 promoter, enhancing FGL1 promoter activity. Lysine-specific demethylase 4A (KDM4A) interacts with Stat3 and facilitates the removal of methyl groups from H3K9me3, thereby enhancing Stat3-mediated transcription of FGL1. Furthermore, we observed that Stat3 and KDM4A promote NSCLC cell proliferation, migration, and invasion partly by upregulating FGL1 expression. Additionally, the expression of FGL1 was significantly higher in cancer tissues (*n* = 90) than in adjacent non-cancerous tissues (n = 90). Furthermore, patients with high FGL1 expression had a shorter overall survival (OS) compared to those with low FGL1 expression. We measured the expression levels of FGL1 on circulating tumor cells (CTCs) in 65 patients and found that patients with a dynamic decrease in FGL1 expression on CTCs exhibited a better therapeutic response. These findings suggest that the dynamic changes in FGL1 expression can serve as a potential biomarker for predicting treatment efficacy in NSCLC. Overall, this study revealed the significant role and regulatory mechanisms of FGL1 in the development of NSCLC, suggesting its potential as a therapeutic target for patients with NSCLC. Future studies should provide more personalized and effective treatment options for patients with NSCLC to improve clinical outcomes.

**Supplementary Information:**

The online version contains supplementary material available at 10.1186/s13046-024-03140-6.

## Introduction

Lung cancer ranks first in terms of mortality rate among all malignancies, accounting for approximately 21% of all cancer-related deaths [1]. Lung cancer can be classified into small cell lung cancer (SCLC) and non-small cell lung cancer (NSCLC) based on its histopathological types, with the latter comprising approximately 85% of all cases [2]. Despite considerable advancements in diagnostic methods, a considerable number of patients are not diagnosed until an advanced stage, resulting in the loss of surgical opportunities [3]. Recent advancements in treatment modalities, particularly the introduction of tyrosine kinase inhibitors (TKIs) [4] and immune checkpoint inhibitors (ICIs), have significantly enhanced the survival of patients with advanced lung cancer [5, 6]. However, the efficacy of targeted therapy and immunotherapy is limited to a specific population of patients [7, 8]. Moreover, drug resistance inevitably leads to treatment failure [9, 10]. Therefore, the exploration of novel therapeutic targets remains a crucial avenue for future efforts.

In 2019, Chen et al*.* published a study in Cell, which highlighted the role of fibrinogen-like protein 1 (FGL1) as a novel immune checkpoint that inhibits T cell activity in a receptor-ligand manner, potentially modulating the resistance to PD-1/PD-L1 therapy [11]. Targeting both PD-L1 and FGL1 can enhance the immune response and anti-cancer effects in lung cancer [12], making FGL1 a promising biomarker for predicting the efficacy of PD-1/PD-L1 therapy as a novel immunotherapy target [13]. Beyond its immunosuppressive role, FGL1 affects tumor processes, such as epithelial-mesenchymal transition (EMT), proliferation, and drug resistance [14–17]. However, the broader biological functions of FGL1 and its role in NSCLC have not been fully elucidated. Therefore, further studies are needed to investigate the biological function of FGL1 in NSCLC. Previous studies on the abnormal expression of FGL1 in tumor tissues have suggested that the IL6/Stat3 signaling pathway, YY1, HNF1a, and other factors may affect the transcription of FGL1 [18–20]. Further studies on the upstream regulatory mechanisms of FGL1 are needed to clarify the reasons behind the abnormal expression of FGL1 in NSCLC.

Epigenetics is the study of chemical modifications and structural changes in chromatin that affect gene expression and cellular function. It includes modifications, like histone methylation and acetylation, with lysine methylation being a key factor regulating gene activity [21–23]. Lysine methylation is the most extensively studied histone modification and is closely associated with the transcriptional activity of target genes [24]. Lysine-specific demethylase 4A (KDM4A) is critically involved in epigenetic regulation, tumor development, and response to treatment. KDM4A typically alters the binding or activity of transcription factors [25]; however, there is limited research on the role of KDM4A in NSCLC. A deeper insight into the regulatory roles of KDM4A in NSCLC can help better understand tumor biology and explore new therapeutic strategies. Stat3 is a critical transcription factor in mammals that is involved in the progression of many cancers, such as NSCLC [26]. Previous studies have shown that Stat3 is closely related to transcriptional regulation and demethylases. Stat3 can recruit histone lysine methyltransferases NSD1 [27], SETD8 [28], and histone demethylase KDM3A [29] to the promoter region of target genes. By dynamically regulating histone methylation, these enzymes an activate the expression of target genes. However, there are no reports on the joint involvement of Stat3 and KDM4A in gene transcription in NSCLC, which deserves further investigation.

Therefore, we measured the co-regulatory effects of KDM4A and Stat3 on FGL1 gene transcription, focusing on upstream regulatory mechanisms increasing FGL1 expression in NSCLC. Using liquid biopsy techniques, we detected FGL1 expression in CTCs from peripheral blood samples of patients with locally advanced or metastatic NSCLC. We found that the dynamic changes in FGL1 expression can potentially serve as a novel biomarker for assessing the therapeutic response in NSCLC. This discovery not only helps understand the malignant nature of NSCLC but also offers a scientific basis for developing potential therapeutic strategies and predicting treatment efficacy.

## Results

### Increased FGL1 expression is associated with poor prognosis of patients with NSCLC

**Fig. 1:**
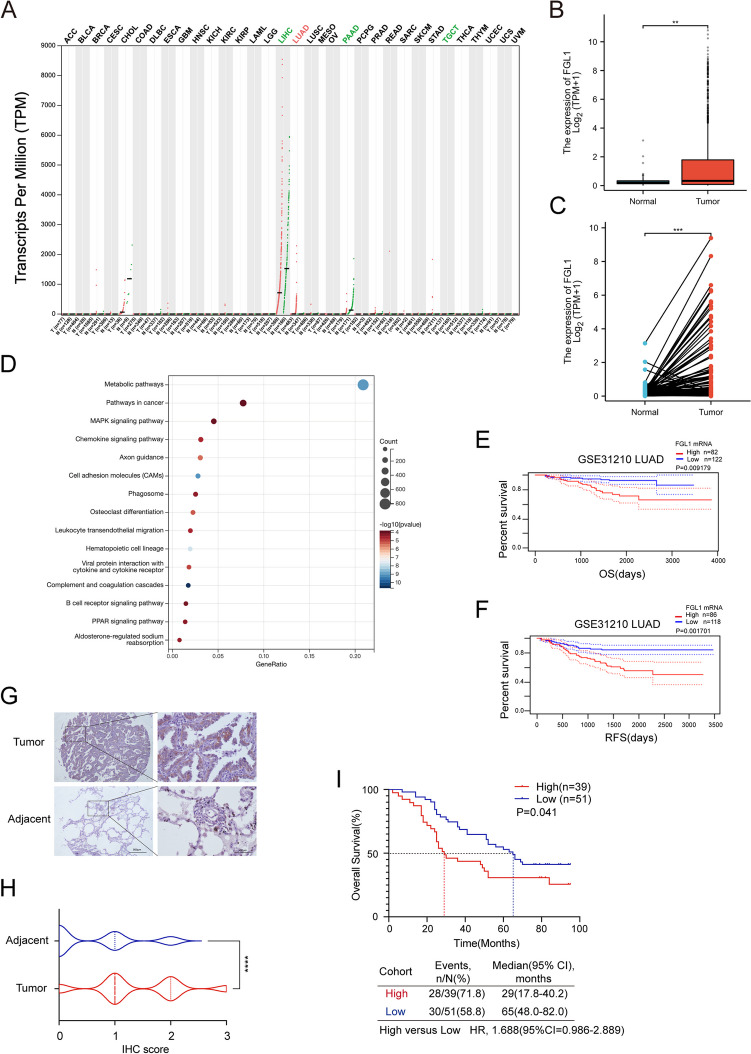
Elevated FGL1 expression was associated with poor prognosis of NSCLC. **A** Pan-cancer analysis of FGL1 expression in cancer and adjacent normal tissues using the GEPIA database. **B** Analysis of FGL1 expression in non-paired samples (*n* = 108 vs. 1041) of TCGA-LUAD LUSC using the TCGA database. Student t-test was used for analysis, and **P < 0.01. **C** Analysis of FGL1 expression in paired samples (*n* = 107) of TCGA-LUAD LUSC using the TCGA database. Student t-test was used for analysis, and ***P < 0.001. **D** Enrichment of FGL1-related gene pathways in the TCGA-LUAD LUSC dataset. **E** Analysis of the correlation between FGL1 and OS using the Prognoscan database (GSE31210). **F** Analysis of the correlation between FGL1 and RFS using the Prognoscan database (GSE31210). **G**, **H** Statistics for the expression of FGL1 in adjacent noncancerous tissues and tumor tissues. Student t-test was used for analysis, and ****P < 0.0001. **I** Survival analysis of the high expression (*n* = 39) and low expression groups (*n* = 51). Note: LUAD refers to lung adenocarcinoma. LUSC refers to squamous cell carcinoma of lung

To investigate FGL1 expression in NSCLC, we analyzed data from the TCGA and GEPIA databases. Our findings demonstrated significantly higher expression of FGL1 in cancer tissues compared to adjacent normal tissues (Fig. 1A-C). Subsequent analysis using the CancerSCEM database [30], revealed high expression of FGL1 in malignant tumor cells and low expression of FGL1 in other cell types (Figure S1A). Analysis of the TCGA-LUAD and TCGA-LUSC dataset highlighted the enrichment of FGL1-related genes in metabolic and tumor-related pathways (Fig. 1D). These findings suggest a potential pro-carcinogenic role for FGL1 in NSCLC. In addition, the PrognoScan database [31] was analyzed to assess the effect of FGL1 on the survival of patients with NSCLC. Our findings indicated that high FGL1 expression is associated with shorter overall survival (OS) and recurrence-free survival (RFS) of patients with LUAD (Fig. 1E-F) and LUSC (Figure S1B-C), although the differences were not statistically significant in LUSC.

To validate our findings from public database analysis, we used NSCLC tissue microarrays and detected the expression of FGL1 protein in cancer tissues and adjacent normal tissues. The results were consistent with those from database analysis, showing that FGL1 expression was significantly higher in cancer tissues compared to adjacent normal tissues *(*Fig. 1G-H*)* (Table 1). Based on tissue microarray immunohistochemistry (IHC) staining of FGL1, patients with “positive” and “strong positive” staining were assigned to the high-expression group, while those with “negative” or “weak positive” staining were assigned to the low-expression group [32] (Fig. S1D). According to the clinical and pathological information and survival of patients, we found a positive association between FGL1 expression and clinicopathological factors. FGL1 promoted lymph node metastasis, leading to cancer progression (Table 2, Fig. S1E-H). Compared to the low expression group, the median OS was significantly shorter in the high FGL1 expression group ((29 months, 95% CI (17.8–40.2) vs. (65 months, 95% CI (48.0–82.0)) *(*F ig. 1I*)*. This finding indicates that increased FGL1 expression is associated with a poor prognosis in patients with NSCLC.

**Table 1:** Expression of FGL1 in adjacent noncancerous and tumor tissues of NSCLC

Groups	Cases (*N* = 180)	FGL1 expression		*P*-value
		Low (*n* = 128)	High *(n* = 52)	
Tumor	90	51	39	*P* <0.0001
Adjacent noncancerous	90	77	13

**Table 2:** Expression of FGL1 in NSCLC samples with clinical characteristics

Variables	Groups	Cases (*n* = 90)	FGL1 low expression (*n* = 51)	FGL1 high expression (*n* = 39)	*P*-value
Univariate analysis					
Gender	Male	48	26	22	0.6727
	Femnale	42	25	17	
Age	≤60	45	26	19	>0.9999
	>60	45	25	20	
Histological stage	I/II	64	37	27	0.8159
	III	26	14	12	
T	T1	51	33	18	0.1916
	T2	29	14	15	
	T3/T4	10	4	6	
N	N0	56	37	19	0.0219^*^
	N1	19	10	9	
	N2/N3	15	4	11	
Clinical stage	I	44	32	12	0.0043^**^
	II	26	13	13	
	III	20	6	14	

T represents tumor size and N represents lymph Node.

In univariate analysis, Chi-square test were used

**P < 0.01

*P < 0.05

### FGL1 knockdown inhibited the proliferation and metastasis of NSCLC

**Fig. 2:**
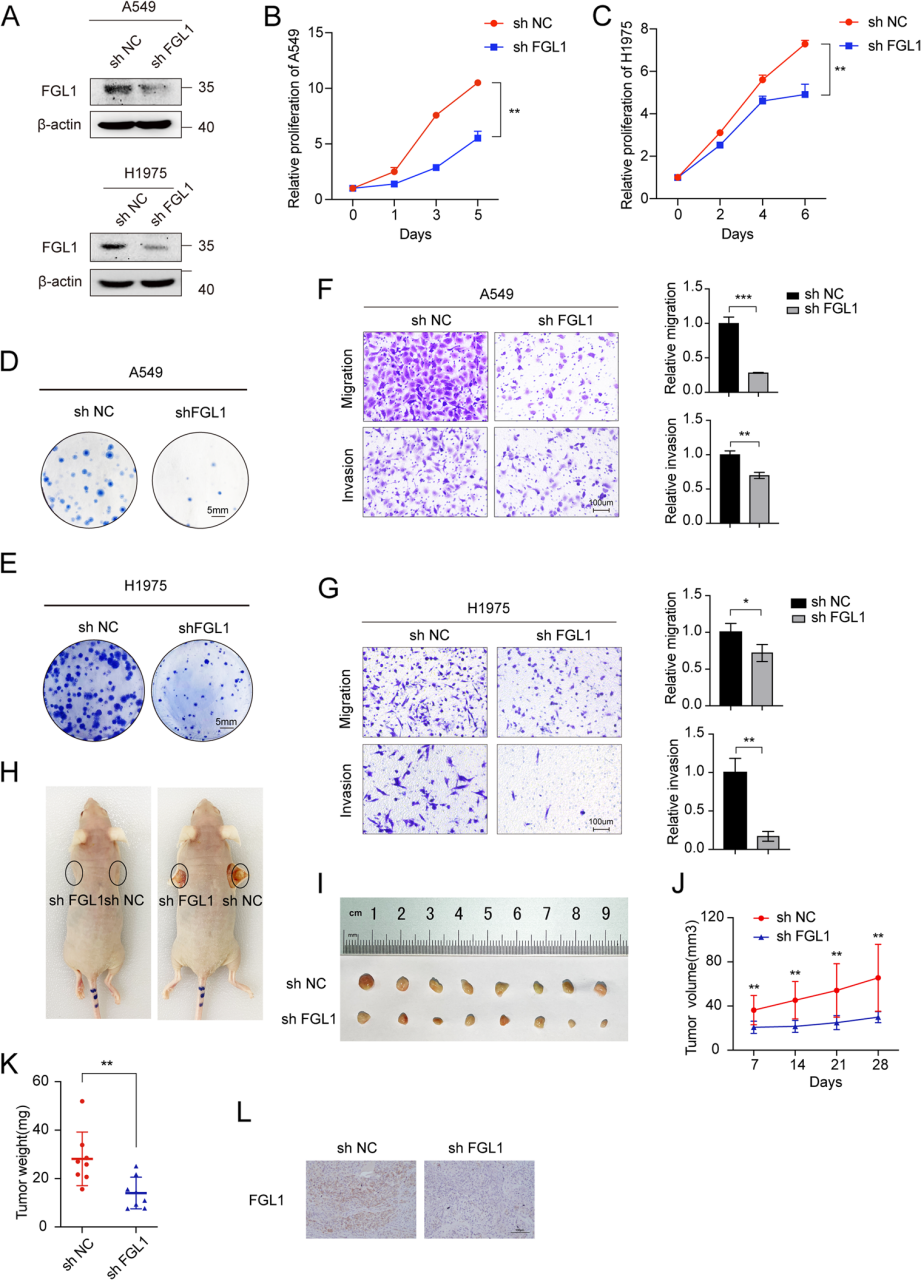
FGL1 knockdown inhibited the proliferation and metastasis of NSCLC. **A** Confirmation of stable knockdown of FGL1 (shFGL1) in A549 and H1975 cell lines induced by lentivirus infection, with scramble shRNA used as a control (shNC). **B**, **C** MTS assay demonstrated that FGL1 knockdown inhibited the proliferation of A549 and H1975 cells. Student t-test was used for analysis, and **P < 0.01. **D**, **E** Colony formation assay showed that FGL1 knockdown suppressed the proliferation of A549 and H1975 cells. Scale bars were 5 mm. **F**, **G** Transwell assay revealed that FGL1 knockdown inhibited the migration and invasion of A549 and H1975 cells (left) and perform statistical analysis(right). Student t-test was used for analysis, and *P < 0.05, **P < 0.01, ***P < 0.001. Scale bars were 100 μm. **H** Representative photos of mice xenograft tumor. Sh NC (right) and sh FGL1 (left) were stably transfected in A549 cells and injected into male mice at 4 weeks old. **I** Xenograft tumors were shown after killing mice. **J** The average tumor volume of sh NC and sh FGL1 was measured every seven days. Bars represent standard alterations of the mean, ** *P* < 0.01. **K** Tumor weights were measured and statistically analyzed using the student’s t-test, **P < 0.01. **L** Immunohistochemical staining of FGL1 in mouse tumor tissues. Scale bars were 100 μm

We conducted phenotype experiments to elucidate the effect of FGL1 on the malignant behavior of NSCLC cells. Among NSCLC cell lines, we found that A549 and H1975 cells showed the greatest difference in FGL1 levels (Fig.  S1I-J). Therefore, stable FGL1 silencing cell lines were established using A549 and H1975 cells as the experimental models (Fig. 2A). MTS assay and colony formation assay were conducted to assess the effect of FGL1 on the proliferative ability of NSCLC cells. Suppression of FGL1 expression reduced colony formation and significantly inhibited the proliferative capacity (Fig. 2B-E). Subsequently, transwell experiments were performed with A549 and H1975 cells, revealing that inhibiting FGL1 expression can suppress cell migration and invasion (Fig. 2F-G). Conversely, overexpression of FGL1 in H1299 cells enhanced the proliferation, migration, and invasion of NSCLC cells (Figure S2).

To explore the in vivo effect of FGL1, an ectopic xenograft tumor model was established using A549 cells infected with lentivirus carrying either sh FGL1 or a negative control lentivirus (sh NC). These findings demonstrated a notable decrease in tumor growth in the FGL1 knockdown group compared to the control group (Fig. 2H-L). Therefore, FGL1 facilitated NSCLC progression by augmenting tumor cell proliferation and metastasis. FGL1 can serve as a novel therapeutic target and biomarker for predicting the therapeutic response in NSCLC, offering encouraging potential for clinical application.

### Stat3 upregulated FGL1 expression via transcriptional regulation

**Fig. 3:**
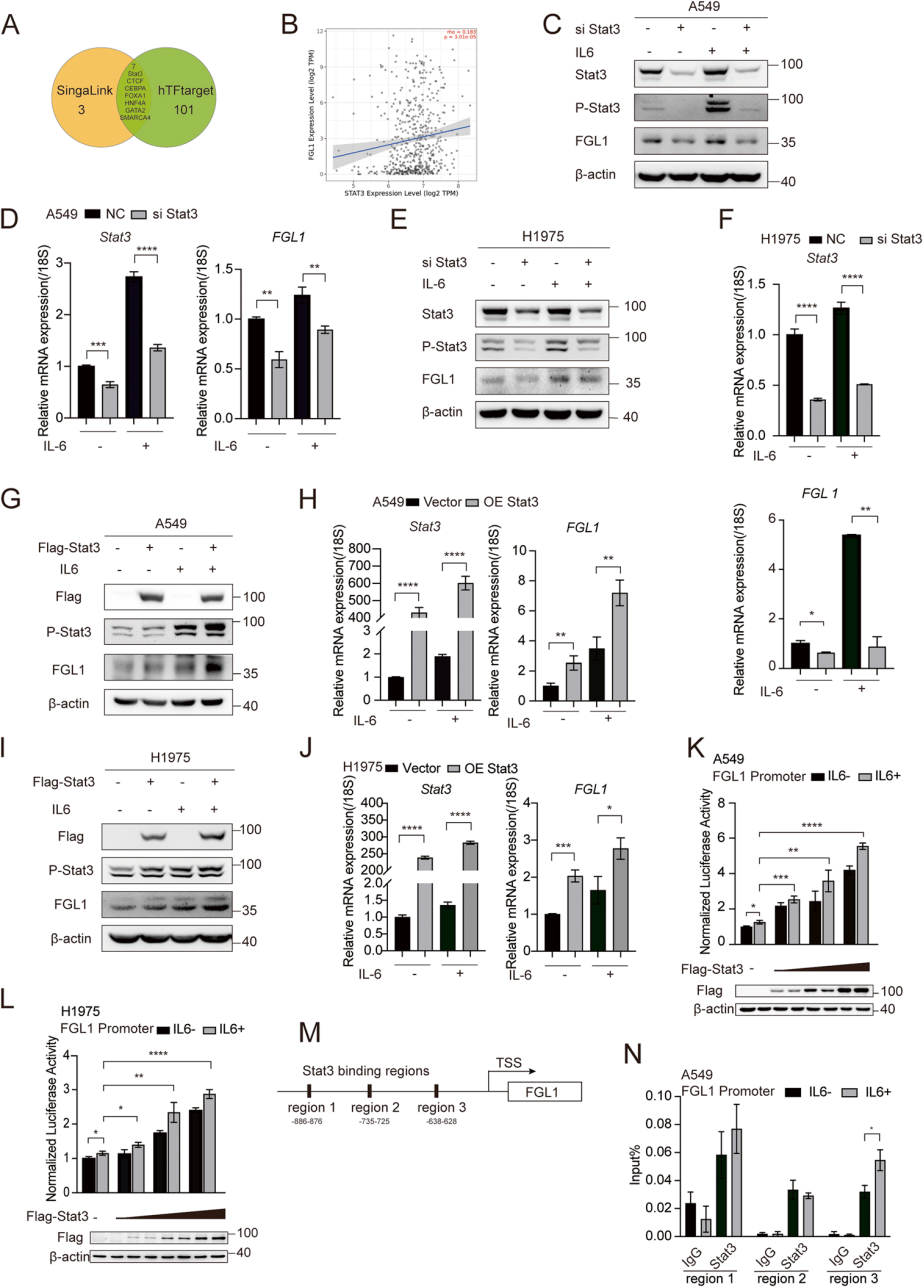
Stat3 upregulated FGL1 expression via transcriptional regulation. **A** Venn diagram showing the proteins predicted to bind to the FGL1 promoter region based on SingaLink and hTFtarget databases. **B** Analysis of the correlation between FGL1 and Stat3 using Timer 2.0. **C**, **D** Effects of Stat3 knockdown on FGL1 expression in A549 cells at the protein (C) and mRNA (D) levels. **E**, **F** Effects of Stat3 knockdown on FGL1 expression in H1975 cells at the protein (E) and mRNA (F) levels. **G**, **H** Effects of Stat3 overexpression on FGL1 expression in A549 cells at the protein (G) and mRNA (H) levels. **I**, **J** Effects of Stat3 overexpression on FGL1 expression in H1975 cells at the protein (I) and mRNA (J) levels. **K**, **L** Gradient overexpression of Stat3 in A549 (K) and H1975 (L) cells and its effect on FGL1 promoter activity. **M** Prediction of potential Stat3 binding regions on the FGL1 promoter using JASPAR. **N** ChIP assay demonstrating the recruitment of Stat3 to three regions on the FGL1 promoter. Student’s t-test was used for analysis, and error bars represent mean ± SD. *P < 0.05, **P < 0.01, ***P < 0.001, ****P < 0.0001

TFs are key regulators of gene expression, directly interpreting the genome and initiating the decoding of DNA sequences. To explore the molecular mechanisms upregulating FGL1 expression, we predicted potential TFs of FGL1 using the SingaLink and hTFtarget databases. Several TFs of FGL1 were identified, including Stat3, CTCF, CEBPA, FOXA1, HNF4A, GATA2, and SMARCA4 (Fig. 3A). Using Timer 2.0 for analysis, Stat3 emerged as an important factor regulating gene transcription in NSCLC, displaying a positive correlation with FGL1 expression [33] (Fig. 3B). The Prognoscan database [31] was utilized to measure the correlation between Stat3 expression and the prognosis of patients with NSCLC (GSE31210). The results revealed that individuals with high Stat3 levels exhibited reduced RFS and OS (Figure S3A). Furthermore, previous studies indicated that IL6 can activate Stat3 to modulate the promoter activity of FGL1 in hepatocellular carcinoma (HCC) [19, 20]. Subsequently, we confirm the association between Stat3 and FGL1 in NSCLC cells. Stat3 knockdown in A549 and H1975 cells reduced both protein and mRNA levels of FGL1 (Fig. 3C-F, S3B). Conversely, the upregulation of Stat3 expression increased the protein and mRNA expression levels of FGL1 (Fig. 3G-J).

Stat3, a member of the STAT family, typically acts as a transcription factor, orchestrating the transcription of target genes, thereby regulating various cancer-related biological processes, such as cell survival, proliferation, angiogenesis, invasion, metastasis, drug resistance, and immune evasion. Thus, we hypothesized that Stat3 may regulate FGL1 expression at the transcriptional level. Subsequently, we conducted a gradient overexpression of Stat3 in A549 and H1975 cells. The results demonstrated that increasing Stat3 expression gradually enhanced the promoter activity of FGL1 (Fig. 3K-L). Thereafter, using JASPAR, we identified three regions on the FGL1 promoter as the binding site of Stat3 (Fig. 3M). We validated this interaction through chip assay [34], which confirmed Stat3 recruitment to these regions on the FGL1 promoter (Fig. 3N). Thus, we proposed that Stat3, as a transcription factor, may enhance the promoter activity of FGL1 and upregulate FGL1 expression.

Given the established role of Stat3 in promoting the growth and metastasis of NSCLC in vivo and in vitro [35], we explored the potential involvement of FGL1 in this process. To this end, we silenced FGL1 in A549 cells. The results revealed that inhibition of FGL1 expression partly attenuated the effects of Stat3 overexpression on tumor cell proliferation, migration, and invasion (Figure S3D-G). In summary, our findings suggest that Stat3 partially promotes NSCLC growth and metastasis by upregulating FGL1 expression.

### KDM4A enhanced the transcriptional activity of Stat3 and upregulated FGL1 expression

**Fig. 4:**
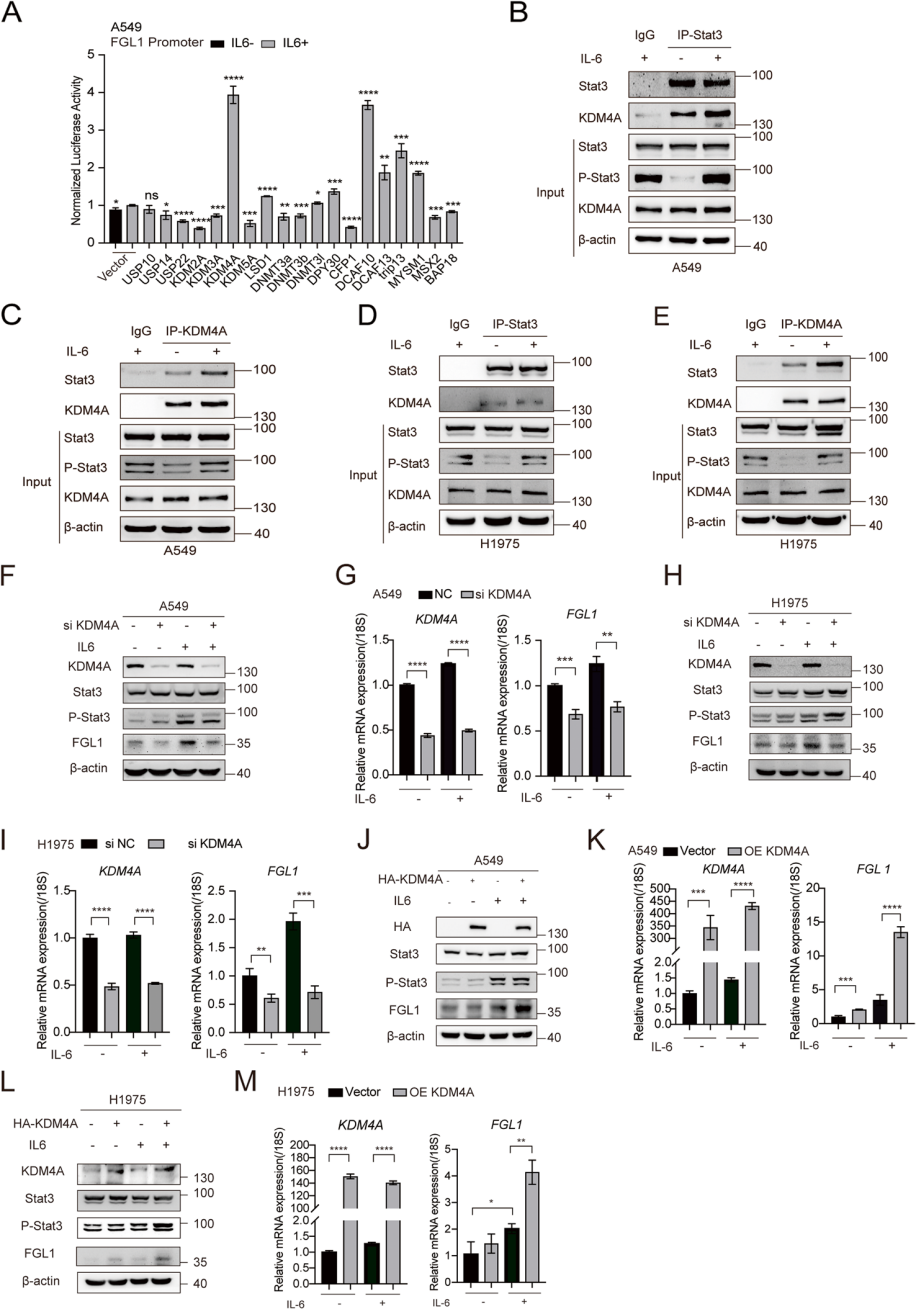
KDM4A and Stat3 interact with each other to promote FGL1 expression. **A** Luciferase assay demonstrating the effect of different cofactors on FGL1 promoter activity. **B**-**E** Co-IP experiments showing the interaction between endogenous Stat3 and KDM4A in A549 (**B**-**C**) and H1975 cells (**D, E**). **F**, **G** KDM4A knockdown and its effect on FGL1 protein (**F**) and mRNA (**G**) expression in A549 cells. **H, I** KDM4A knockdown and its effect on FGL1 protein (**H**) and mRNA (**I**) expression in H1975 cells. **J**, **K** KDM4A overexpression and its effect on FGL1 protein (**J**) and mRNA (**K**) expression in A549 cells. **L**, **M** KDM4A overexpression and its effect on FGL1 protein (**L**) and mRNA (**M**) expression in H1975 cells. Student’s t-test was used for analysis, and error bars represent mean ± SD. *P < 0.05, **P < 0.01, ***P < 0.001, ****P < 0.0001 and ns stands for no significance

TFs typically do not regulate target gene transcription alone. They often engage cofactors that either augment or inhibit the transcriptional activity of TFs on the target gene. Hence, we proceeded to explore the potential factors enhancing the promoter activity of FGL1. We overexpressed Stat3 and various cofactors in A549 cells, and the results showed that several cofactors can alter the activity of the FGL1 promoter when Stat3 is overexpressed. Among them, the promoting effect of KDM4A was the most prominent (Fig. 4A). Consistently, immunoprecipitation experiments indicated that endogenous and exogenous KDM4A and Stat3 interact with each other (Fig. 4B-E, S3H). Furthermore, immunofluorescence experiments confirmed that under IL6 stimulation, KDM4A and Stat3 colocalize in the cell nucleus (Figure S3I). Subsequent knockdown of KDM4A downregulated the transcriptional activity of Stat3 on the FGL1 promoter region (Figure S3J). We proceeded to confirm the relationship between KDM4A and FGL1 expression. Silencing KDM4A in A549 and H1975 cells reduced both mRNA and protein levels of FGL1 (Fig. 4F-I). In contrast, KDM4A overexpression increased both mRNA and protein levels of FGL1 (Fig. 4J-M). These findings support the notion that KDM4A interacts with Stat3 in transcriptional regulation, thereby enhancing FGL1 promoter activity and upregulating FGL1 expression. To explore whether the correlation between Stat3 and KDM4A is specific to FGL1, we measured the expression of other target genes of Stat3. The results showed that KDM4A knockdown decreased the expression of other target genes of Stat3 (Figure S4A). Therefore, we concluded that KDM4A can bind to Stat3 and upregulate its transcriptional activity, and this effect was not specific to FGL1.

### KDM4A demethylated H3K9me3 and upregulated the transcriptional activity of Stat3

**Fig. 5:**
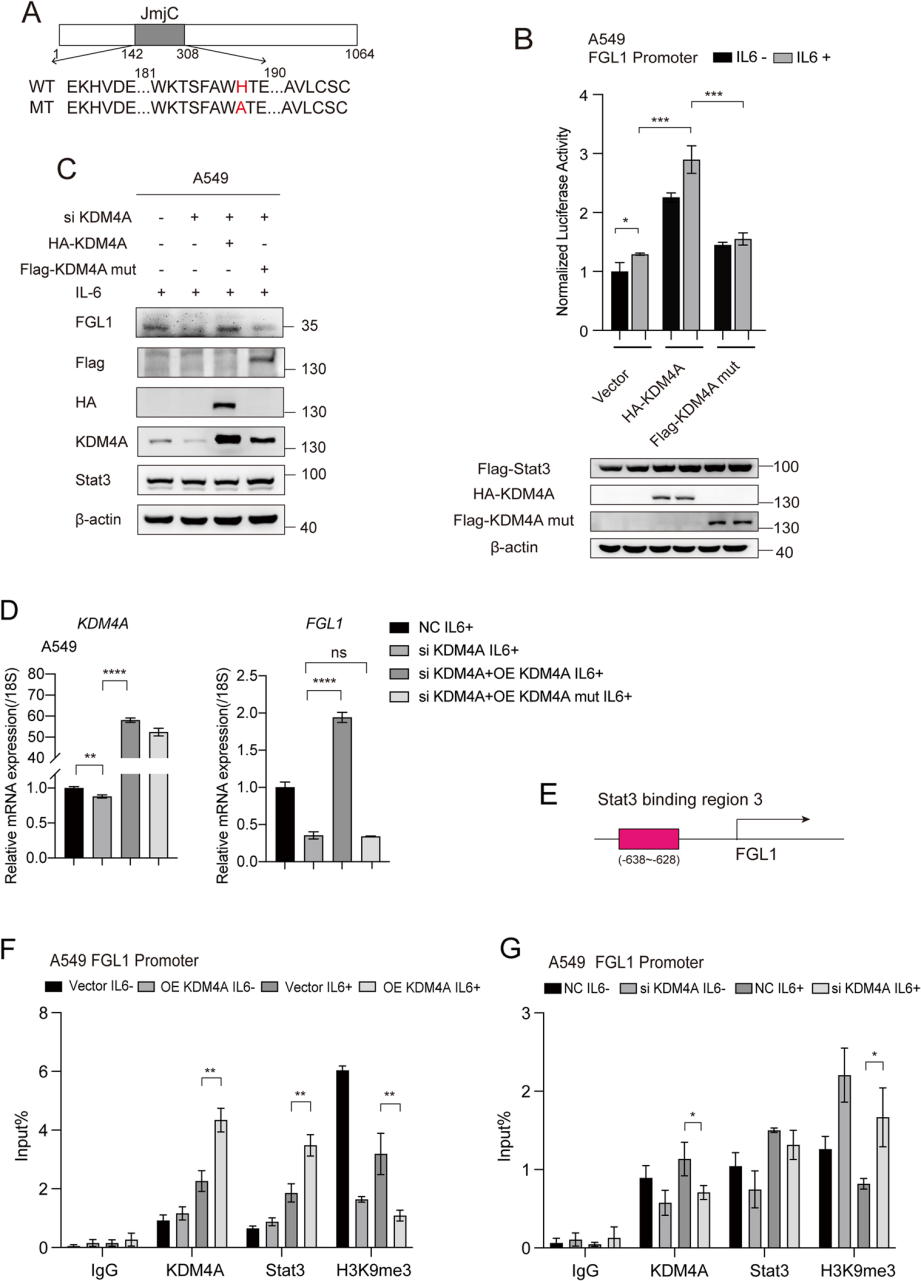
KDM4A upregulated FGL1 promoter activity through its enzymatic activity. Representation of KDM4A wt and KDM4A mut (loss of function mutation in KDM4A demethylase activity) in a diagram. **B** Effect of KDM4A wt and KDM4A mut on FGL1 promoter activity. **C**, **D** Effect of KDM4A wt and KDM4A mut on FGL1 protein (C) and mRNA (D) levels. **E** Stat3 binding region 3 on the FGL1 promoter. **F**, **G** Chip assay demonstrating the effects of KDM4A overexpression (**F**) or knockdown (**G**) on H3K9me3 levels and Stat3 recruitment at the FGL1 promoter. Student’s t-test was used for analysis, and error bars represent mean ± SD. *P < 0.05, **P < 0.01, ***P < 0.001, ****P < 0.0001 and ns stands for no significance

We further investigate whether the demethylase activity is required for modulation function of KDM4A on FGL1 promoter activity. The results showed that KDM4A H188A had almost no effect FGL1 promoter activity, compared with the enhancement of FGL1 promoter activity mediated by KDM4A wt (Fig. 5A-B). KDM4A knockdown reduced FGL1 expression, while KDM4A overexpression restored it, and the enzymatic activity mutant did not allow restoring FGL1 expression (Fig. 5C-D). These results indicate that KDM4A modulates FGL1 expression via its demethylase activity. Furthermore, we verified the recruitment of KDM4A to Stat3-binding regions within the FGL1 promoter. The results showed that KDM4A can be recruited to the FGL1 promoter and align with Stat3 recruitment (Figure S4B). We validated histone modifications on the FGL1 promoter. KDM4A overexpression reduced the H3K9me3 level and KDM4A knockdown increased the H3K9me3 level on the FGL1 promoter, suggesting that KDM4A upregulates FGL1 by demethylating H3K9me3 (Fig. 5E-G).

Next, we verified that the transcriptional activity of FGL1 was stronger in the presence of both KDM4A and Stat3 than in the presence of only one of them (Figure S4C-D). Conversely, Stat3 knockdown attenuated the promoting effect of KDM4A on FGL1 expression (Figure S4E-F), suggesting that KDM4A upregulates FGL1 via Stat3.

### KDM4A partly promoted NSCLC proliferation and metastasis by upregulating FGL1

**Fig. 6:**
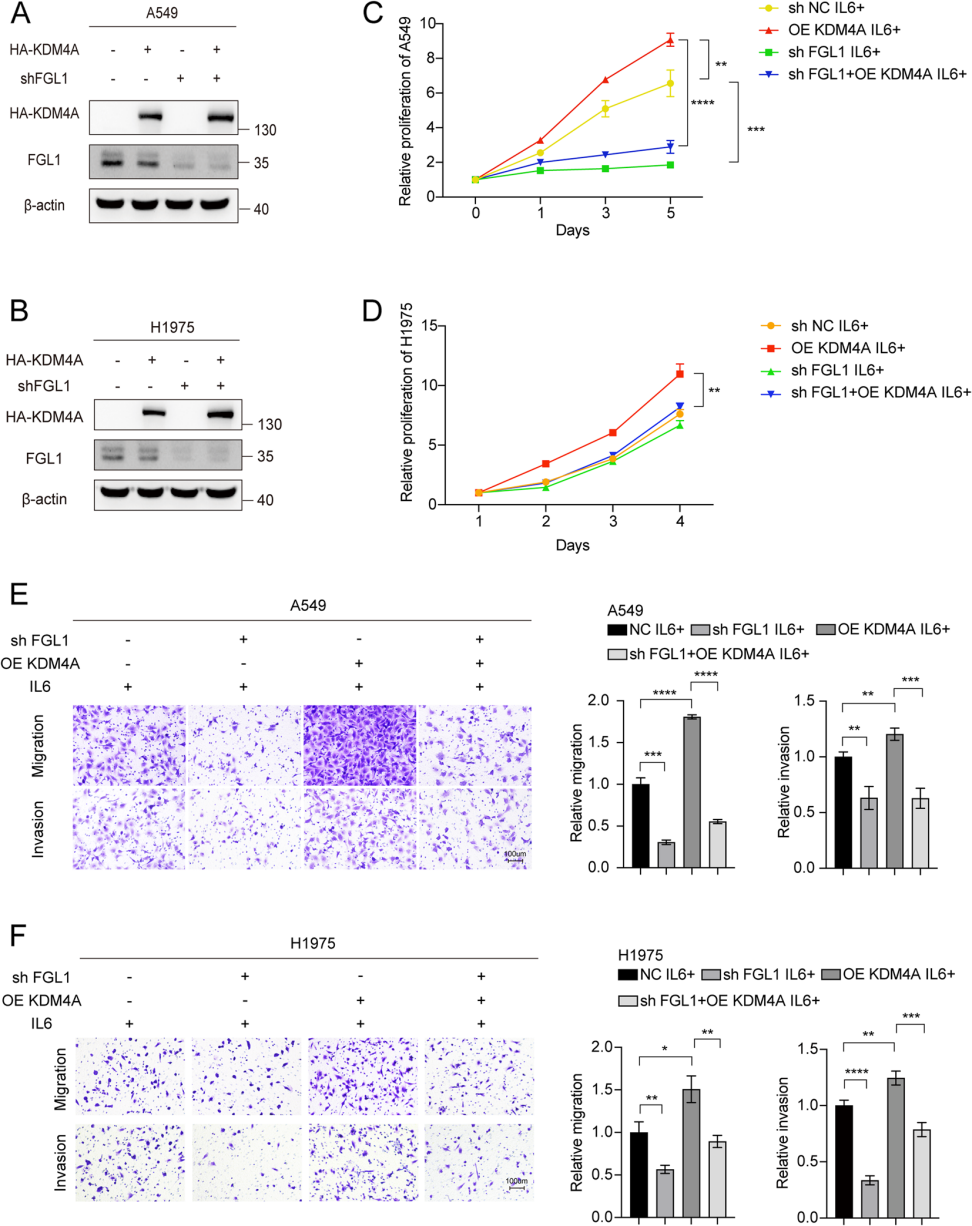
KDM4A partly promoted the proliferation, migration, and invasion of NSCLC cells by upregulating FGL1. **A**, **B** Validating the protein expression of HA-KDM4A and FGL1 under different treatment conditions in A549 cells (**A**) and H1975 cells (**B**). **C**, **D** MTS assay to assess the effect of FGL1 knockdown and overexpression on the proliferation of A549 cells (**C**) and H1975 cells (**D**). **E**, **F** Transwell assay to determine the effect of FGL1 knockdown and overexpression on cell migration and invasion. Student’s t-test was used, and error bars represent mean ± SD. *P < 0.05, **P < 0.01, ***P < 0.001, ****P < 0.0001. Scale bars were 100 μm

In KM-plotter analysis [36] of patients with lung adenocarcinoma, high expression of KDM4A was associated with shorter overall survival (Figure S5A). Subsequently, KDM4A knockdown in A549 and H1975 cells inhibited cell proliferation, migration, and invasion (Figure S5B-E). Conversely, KDM4A overexpression promoted cell proliferation, migration, and invasion in NSCLC cells (Figure S5F-I). The proliferative and migratory effects of KDM4A were enhanced in the presence of IL6, which enhanced the transcriptional regulatory role of Stat3 (Figure S5 B- I). FGL1 knockdown partly reversed the promoting effect of KDM4A on the proliferation, migration, and invasion of NSCLC cells (Fig. 6). These results indicate that KDM4A promotes cell proliferation, migration, and invasion in NSCLC cells partly by upregulating FGL1 expression.

**Fig. 7:**
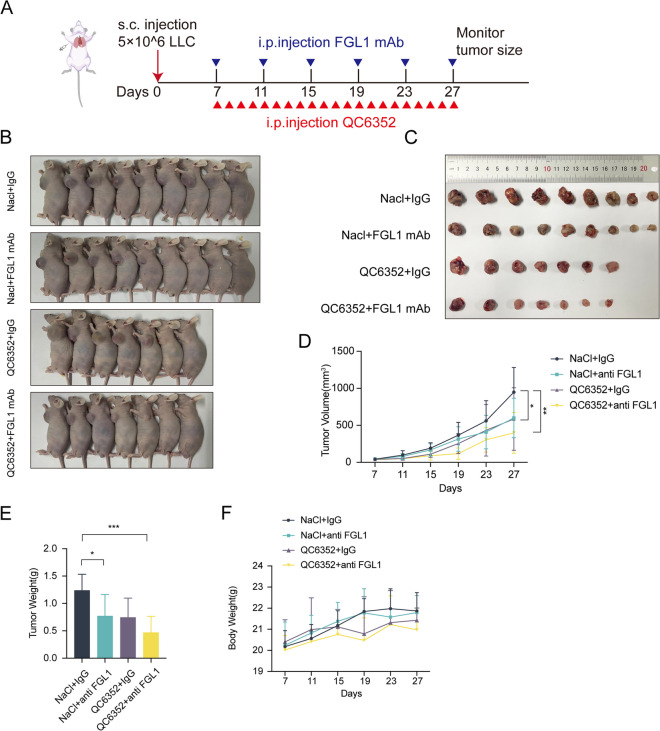
KDM4A inhibition confers tumor sensitivity to FGL1 mAb. **A** Schematic representation of the animal experiment process. Mice bearing LLC syngeneic tumors were treated for 21 days with QC6352 and (or) FGL1 mAb. **B**, **C** Photos of mice tumor. **D** The average tumor volume was measured every four days. Bars represent standard alterations of the mean, *P < 0.05, ** *P* < 0.01. **E** Tumor weights were measured and statistically analyzed using the student’s t-test, *P < 0.05, ***P < 0.001. **F** Mice weights were measured

To explore the in vivo effect of KDM4A, a syngeneic model was established using LLC cells. Then, treatment with KDM4A inhibitor (QC6352) at a dose of 10 mg/kg alone or in combination with FGL1 mAb was administered to tumor-bearing mice (Fig. 7A). The results confirmed that the application of QC6352 significantly inhibited tumor growth, and the combined treatment with FGL1 mAb showed an even more pronounced inhibitory effect on the tumor (Fig. 7B-E). Furthermore, the mice exhibited good tolerance to the combined treatment, with no significant changes in body weight (Fig. 7F). These above data further indicated that KDM4A partly promoted proliferation and metastasis through upregulating FGL1 in NSCLC.

### FGL1 predicted therapeutic efficacy in NSCLC

**Fig. 8:**
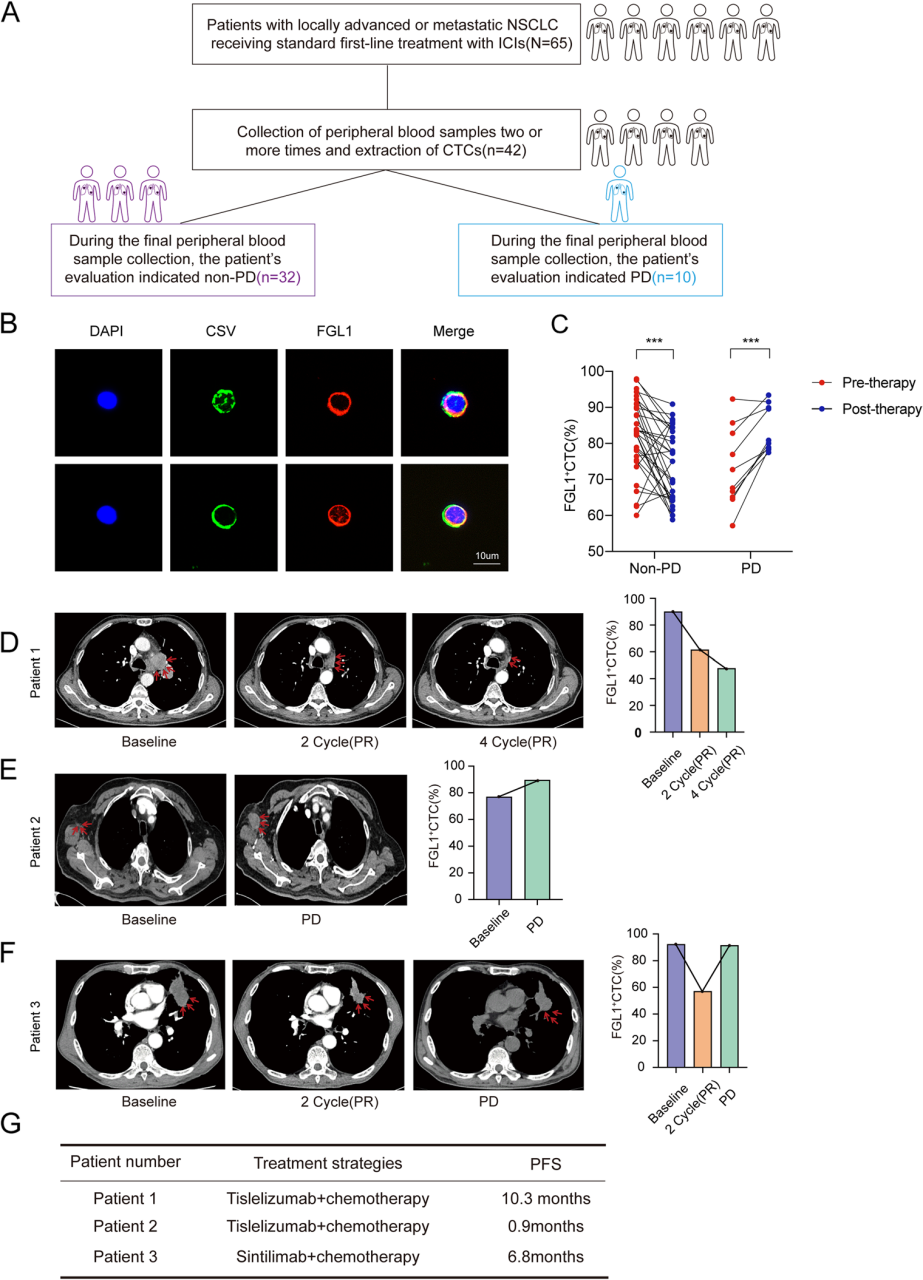
FGL1 is a potential biomarker for efficacy in NSCLC based on CTC detection. **A** The flowchart showing the 65 included patients. Peripheral blood samples were collected from 42 patients two or more times to assess the dynamic changes in FGL1 expression. As of January 31, 2024, 32 patients showed a non-PD response, while 10 patients showed a PD. **B** The distribution of CSV and FGL1 by immunofluorescence staining in CTCs. Cells were stained with anti-DAPI (Blue), anti-CSV (Green), and anti-FGL1 (Red). Scale bars were 10 μm. **C** The expression of FGL1 on CTCs before and after treatment in both non-PD and PD groups. Student t-test was used for analysis, and ***P < 0.001. **D**-**F** The results of CT (left) and the dynamic changes in FGL1 + CTCs in peripheral blood (right) of patients in the non-PD (**D**) and PD (**E**, **F**) groups. Detailed patients’ information is presented in the supplementary data. **G** The table displays the treatment regimens and PFS for the 3 patients in Fig. 8 D-F

**Fig. 9:**
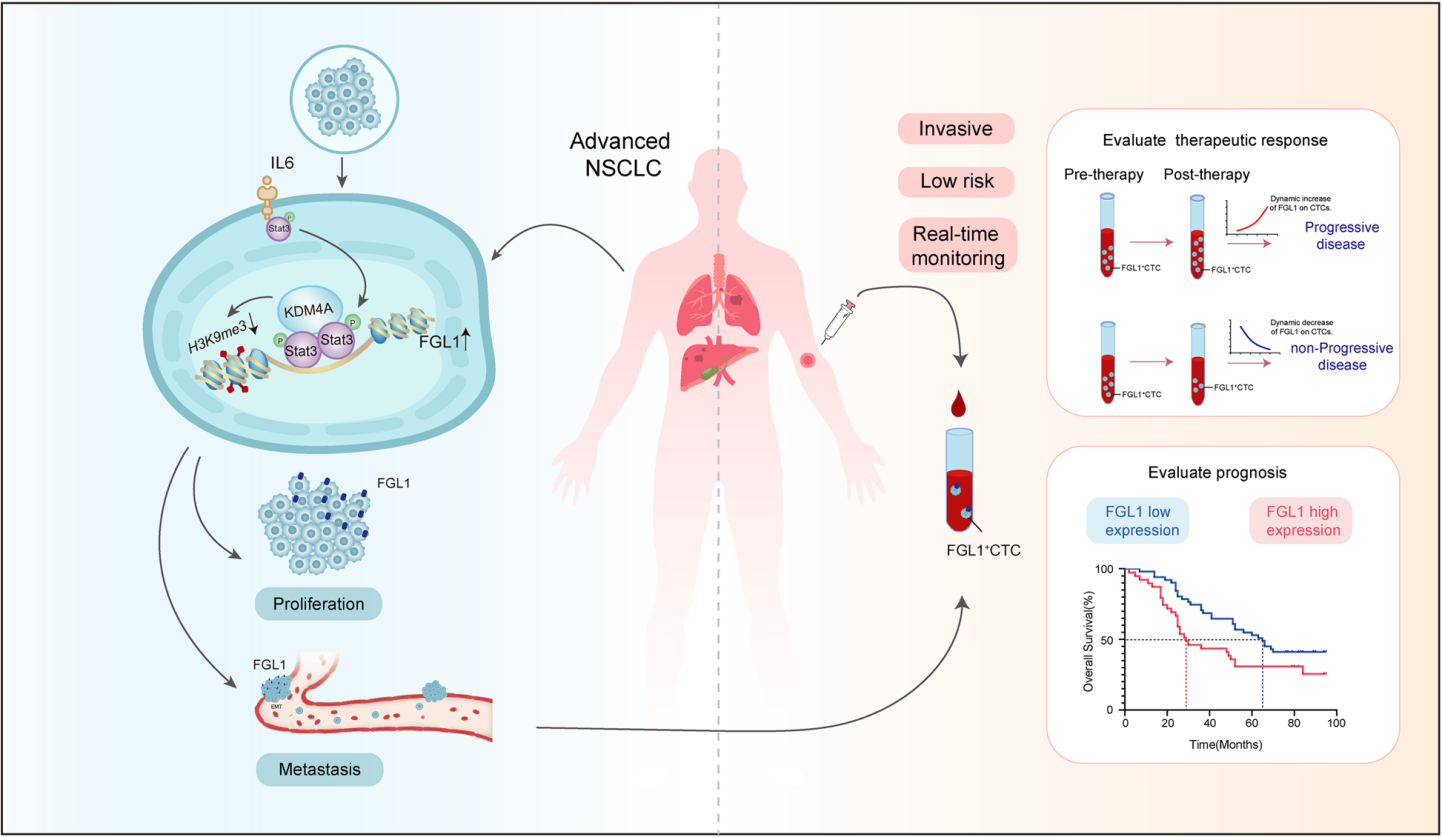
Schematic diagram of the upstream regulatory mechanism and clinical role of FGL1. KDM4A, as a histone demethylase, interacts with Stat3 in the promoter region of FGL1, demethylating H3K9me3 to upregulate the activity of the FGL1 promoter and enhance FGL1 expression. High expression of FGL1 in NSCLC promotes tumor growth and metastasis, and patients with high FGL1 expression have a relatively poor prognosis. Liquid biopsy monitors changes in FGL1 expression on CTCs in a relatively non-invasive, low-risk, and real-time manner. Increased FGL1 expression indicates disease progression, while decreased FGL1 expression suggests disease stability or remission

We collected peripheral blood samples from 65 patients with locally advanced or metastatic NSCLC. The baseline characteristics of patients are shown in the Table 3. All patients received standard first-line treatment with ICIs, and an efficacy assessment was performed every two cycles. Non-progressive disease (Non-PD) included patients with CR, PR, and SD. In total, peripheral blood samples were collected from 42 patients two or more times. As of January 31, 2024, 32 patients remained in the non-PD state, while 10 patients showed progressive disease (PD) (Fig. 8A). CTCs were extracted from the peripheral blood and subjected to immunofluorescence staining [37]. The results demonstrated that FGL1 could be detected in CTCs in the peripheral blood of all patients (Fig. 8B). Compared to baseline, patients in the non-PD group exhibited a significant decrease in FGL1 expression on CTCs. On the other hand, patients in the PD group showed a significant increase in FGL1 expression on CTCs compared to baseline. These differences were statistically significant (Fig. 8C). More importantly, we found that as treatment progressed, the expression of FGL1 gradually decreased in the non-PD group (Figure S6A). In contrast, in the PD group, patients exhibited a significant increase in FGL1 levels with the progression of the disease compared to both baseline and the 2-cycle time point (Figure S6B). The imaging results show that patient 1, who received first-line treatment based on tislelizumab, achieved a good response during treatment. As the proportion of FGL1^+^CTCs decreased, the patient showed a PR in tumor assessment (PFS = 10.3 months) (Fig. 8D). Whereas patient 2, who received first-line treatment based on tislelizumab, showed an increase in the proportion of FGL1^+^CTCs during treatment. As a result, the tumor assessment indicated PD (PFS = 0.9 months) (Fig. 8E). Patient 3 received first-line treatment based on Sintilimab. After 2 cycles, there was a decrease in the proportion of FGL1^+^ CTCs, leading to a reduction in lung lesions. Subsequently, the proportion of FGL1^+^ CTCs increased, resulting in a tumor assessment of PD (PFS = 6.8 months) (Fig. 8F). In addition, we found that in CTCs, the expression of KDM4A or Stat3 is positively correlated with FGL1 (Figure S6D). These findings indicate that FGL1 is closely associated with the prognosis of NSCLC and may serve as a biomarker for predicting NSCLC progression after treatment Fig. 9.

**Table 3:** Baseline characteristics of the patients

Variables	Groups	Cases (*N* = 65)
Gender	Male	53
	Female	12
Age	<65	30
	≥65	35
ECOG score standard	0-1	61
	2	4
Smoking status	Smoking	43
	No smoking	22
Clinical stage	III	30
	IV	35
Pathological type	Squamous carcinoma	28
	Adenocarcinoma	37
Subsequent antitumor therapy	Immunotherapy+Chemotherapy	54
	Immunotherapy+Chemotherapy+Bevacizumab	11

## Discussion

In recent years, novel treatment approaches, including immunotherapy and targeted therapy, have been employed for lung cancer. Despite the evolution of treatment concepts and strategies, patients with NSCLC continue to face challenges such as drug resistance, recurrence, and metastasis. Therefore, identifying new biomarkers for predicting treatment efficacy and discovering therapeutic targets is crucial for achieving precision treatment, overcoming drug resistance, improving prognosis, and enhancing the quality life of patients. Our study revealed a significant increase in FGL1 expression in NSCLC, suggesting that FGL1 could serve as both a novel biomarker for immunotherapy and a therapeutic target in NSCLC, with substantial potential for clinical translation. Thus, a detailed understanding of the molecular mechanisms regulating FGL1 expression is essential for developing new therapeutic strategies for NSCLC.

FGL1 is a proliferation- and metabolism-related factor secreted by the liver. It was shown to be abnormally expressed in various tumors [13]. However, the upstream regulatory mechanisms leading to its abnormal expression remains unclear. Previous studies have shown that IL6 and Stat3 promote FGL1 expression but have not elucidated their detailed molecular mechanisms [19, 20, 38]. Our research found that in NSCLC cells, Stat3 can be recruited directly to the FGL1 promoter region, enhancing FGL1 promoter activity and thereby promoting FGL1 expression. Epigenetic regulation, which plays a crucial role in tumorigenesis, refers to the mechanism by which cell function and development are regulated at the gene expression level without changing the DNA sequence. In this study, we are surprised to find that the epigenetic enzyme KDM4A serves as a key co-regulator of Stat3 by demethylating H3K9me3 on the FGL1 promoter region, enhancing FGL1 promoter activity. Most importantly, KDM4A enhances not only the transcription of FGL1 but also the expression of other Stat3 target genes. Our discovery fills the gap in understanding how epigenetic modifications regulate FGL1 gene transcription and enhances our knowledge of the crucial role of KDM4A in the malignant processes of NSCLC. In terms of clinical applications, considering the important role of KDM4A in tumor development, small molecule inhibitors targeting KDM4A have been developed [39]. However, due to the broad range of downstream molecules regulated by KDM4A and the significant roles they play, these inhibitors may have unavoidable side effects and their effectiveness may be unsatisfactory. Our research indicates that FGL1, as one of the downstream molecules of KDM4A, plays a crucial role in the progression of NSCLC. This suggests that future development of drugs targeting FGL1 instead of KDM4A might provide greater benefits for NSCLC patients.

In addition to these advancements, our study has explored the clinical translational potential of FGL1 using a novel circulating tumor cells (CTCs) platform. As part of liquid biopsy, CTCs offer a non-invasive, simple, and minimally invasive diagnostic approach, crucial for early detection, prognosis, and treatment monitoring in cancer [40]. However, detecting CTCs is challenging due to their rarity and heterogeneity. Current methods like the CellSearch system, which targets epithelial cell adhesion molecules (EpCAM), are less effective for capturing mesenchymal-type CTCs common in NSCLC [41, 42]. Studies have shown that cell surface vimentin (CSV) is more efficient than EpCAM in capturing CTCs in solid tumors, including lung cancer [19, 43–45]. Additionally, we found that the expression level of CSV is universally high in NSCLC cell lines (Figure S6C). Therefore, we utilized a novel CTC detection platform, the CSV enrichment technique, to detect CTCs and measure the expression of FGL1 on CTCs of patients with locally advanced and metastatic NSCLC. FGL1 was detectable on CTCs in all NSCLC patients, and its dynamic changes can indicate treatment response. This method non-invasively provides tumor-related information, suggesting that FGL1 has significant clinical potential both as a therapeutic target and as a biomarker for efficacy and prognosis in NSCLC.

In summary, our results indicate that FGL1 is abnormally overexpressed in NSCLC tissues, promoting the malignant behavior of NSCLC cells. KDM4A, a histone demethylase, acts as a novel coactivator of the Stat3 signaling pathway, activating the FGL1 promoter and increasing the expression of FGL1 and other Stat3 target genes. FGL1 expression on CTCs allows for non-invasive, real-time monitoring of therapeutic efficacy and prognosis, and serves as a potential target for precision therapy in NSCLC. Collectively, our findings provide a strong theoretical basis for developing new therapeutic strategies targeting FGL1 in NSCLC.

### Supplementary Information

Supplementary Material 1. Fig. S1. (A) Cell component comparison between single-cell samples in NSCLC using the CancerSCEM database. (B) Analysis of the correlation between FGL1 and OS using the Prognoscan database (GSE17710). (C) Analysis of the correlation between FGL1 and RFS using the Prognoscan database (GSE17710). (D) Scoring based on the intensity of FGL1 expression: 0 points for "negative", 1 point for "weak positive", 2 points for "positive", and 3 points for "strong positive". (E) Representative images of tissue slices from patients with lymph node stages of N0, N1, and N2. (F) According to lymph node staging, the expression of FGL1 was analyzed in 98 patients with cancer. **P < 0.01. (G) Representative images of tissue slices from patients with clinical stages I, II, and III. (H) According to the clinical staging, the expression of FGL1 was analyzed in 98 patients with cancer. *P < 0.05, **P < 0.01. (I) The expression of FGL1 protein was measured on five NSCLC cancer cell lines (A549, H1299, H1975, H460, and SPCA1). (J) The expression of FGL1 mRNA was measured on five NSCLC cancer cell lines (A549, H1299, H1975, H460, and SPCA1).

Supplementary Material 2. Fig. S2. (A) Validation of FGL1 protein expression after FGL1 knockdown using siRNA in A549 cells and FGL1 overexpression in H1299 cells. (B) MTS assay to assess the effect of FGL1 knockdown on the proliferation of A549 cells and FGL1 overexpression on the proliferation of H1299 cells. (C) Clonogenic assay for evaluating the effect of FGL1 knockdown on the proliferation of A549 cells and FGL1 overexpression on the proliferation of H1299 cells. Scale bars were 5 mm. (D) Transwell assay to determine the effect of FGL1 knockdown on the migration and invasion of A549 cells and FGL1 overexpression on the migration and invasion of H1299 cells. Scale bars were 100 μm. (E) Scratch assay to measure the effect of FGL1 knockdown on the migration of A549 cells and FGL1 overexpression on the migration of H1299 cells. The images are magnified by 100 times. Student’s t-test was used for analysis and error bars represent mean ± SD. *P < 0.05, **P < 0.01, ***P < 0.001, ****P < 0.0001.

Supplementary Material 3. Fig. S3. (A) Analysis of the GSE31210 dataset to assess the effect of Stat3 expression on OS and RFS in patients with LUAD. (B) The effect of IL6 stimulation at different time points and concentrations on Stat3 phosphorylation and FGL1 protein expression. (C) Validation of Flag-Stat3 and FGL1 protein expression in cells under different treatment conditions. (D) MTS assay to assess the effect of FGL1 knockdown and Stat3 overexpression on cell proliferation. Scale bars were 100 μm. (E) Colony formation assay to validate the effect of FGL1 knockdown and Stat3 overexpression on cell proliferation. Scale bars were 5 mm. (F) Transwell assay to determine the effect of FGL1 knockdown and Stat3 overexpression on cell migration and invasion. Scale bars were 100 μm. (G) Statistical analysis of Figure D. Student’s t-test was used for analysis and error bars represent mean ± SD. **P < 0.01, ***P < 0.001. (H) Co-IP experiments showing the interaction between exogenous Stat3 and KDM4A in HEK-293 cells. (I) The distribution of KDM4A and Stat3 with or without IL6 in immunofluorescence staining of A549 cells. Cells were stained with anti-DAPI (Blue), anti-Stat3 (Red), and anti-KDM4A (Green). Scale bars are 20 μm. (J) Luciferase assay demonstrating the effect of KDM4A on FGL1 promoter activity. Student’s t-test was used for analysis and error bars represent mean ± SD. **P < 0.01, ***P < 0.001, ****P < 0.0001. Scale bars were 100 μm.

Supplementary Material 4. Fig. S4. (A) Effect of KDM4A knockdown on the mRNA expression of classical target genes of Stat3. (B) Recruitment of KDM4A to Stat3 binding regions on the FGL1 promoter. (C-D) Changes in FGL1 promoter activity under different treatment conditions in A549 (C) and H1975 cells (D). (E–F) Changes in FGL1 protein (E) and mRNA (F) expression upon stable Stat3 knockdown and simultaneous overexpression of KDM4A in A549 cells. Student’s t-test was used for analysis, and error bars represent mean ± SD. *P < 0.05, **P < 0.01, ***P < 0.001, ****P < 0.0001, and ns stands for no significance.

Supplementary Material 5. Fig. S5. (A) Analysis of KDM4A expression and its effect on OS in LUAD patients using the KM plotter. (B) Validation of KDM4A protein expression under different treatment conditions in A549 and H1975 cells. (C) MTS assay exhibiting the effect of KDM4A knockdown on A549 and H1975 cell proliferation. (D-E) Transwell assay showing the effect of KDM4A knockdown on the migration and invasion of A549 and H1975 cells. Scale bars were 100 μm. (F) Validation of KDM4A protein expression under different treatment conditions in A549 and H1975 cells. (G) MTS assay exhibiting the effect of KDM4A overexpression on the proliferation of A549 and H1975 cells. (H) Transwell assay illustrating the effect of KDM4A overexpression on the migration and invasion of A549 cells. Scale bars were 100 μm. (I) Transwell assay displaying the effect of KDM4A overexpression on the migration and invasion of H1975 cells. Student’s t-test was used for analysis, and error bars represent mean ± SD. *P < 0.05, **P < 0.01, ***P < 0.001, ****P < 0.0001. Scale bars were 100 μm.

Supplementary Material 6. Fig. S6. (A) Changes in FGL1 expression on CTCs from patients who showed a non-PD response after 2 and 4 cycles of evaluation. Student’s t-test was used for analysis, and error bars represent mean ± SD. *P < 0.05, **P < 0.01, ***P < 0.001. (B) Changes in FGL1 expression on CTCs from patients who showed PD at the final assessment. (C) Bar graphs showing the expression levels of CSV in NSCLC cell lines. (D) The distribution of KDM4A、Stat3、CSV and FGL1 by immunofluorescence staining in CTCs. Cells were stained with anti-DAPI (Blue), anti-Stat3 and anti-CSV(Green), anti-KDM4A and anti-FGL1 (Red). Scale bars were 20 μm.

Supplementary Material 7.

## Data Availability

All the data supporting the conclusions in this article are presented in this article and its additional files.

## Abbreviations

NSCLC: Non-Small Cell Lung Cancer
SCLC: Small Cell Lung Cancer
CTCs: CTCs: Circulating tumor cells
TKIs: Tyrosine kinase inhibitors
ICIs: Immune checkpoint inhibitors
CR: Complete Response
PR: Partial Response
PD: Progression disease
Non-PD: Non-progressive disease
SD: Stable disease
EMT: Epithelial-mesenchymal transition
CSV: Cell surface vimentin
EpCAM: Epithelial cell adhesion molecules
RECIST: Response Evaluation Criteria in Solid Tumors
PFS: Progression-free survival
OS: Overall survival
DFS: Disease-free survival
PBMC: Peripheral blood mononuclear cell
FBS: Fetal bovine serum
IHC: Immunohistochemistry

## References

[1] Bray F, Laversanne M, Sung H, et al. Global cancer statistics 2022: GLOBOCAN estimates of incidence and mortality worldwide for 36 cancers in 185 countries [J]. CA Cancer J Clin. 2024;74(3):229–63. 10.3322/caac.21834 38572751

[2] Herbst RS, Morgensztern D, Boshoff C. The biology and management of non-small cell lung cancer [J]. Nature. 2018;553(7689):446–54. 29364287 10.1038/nature25183

[3] Blandin Knight S, Crosbie P A, Balata H, et al. Progress and prospects of early detection in lung cancer [J]. Open Biol. 2017;7(9):170070. 10.1098/rsob.170070 PMC5627048 28878044

[4] Zhou C, Wu YL, Chen G, et al. Erlotinib versus chemotherapy as first-line treatment for patients with advanced EGFR mutation-positive non-small-cell lung cancer (OPTIMAL, CTONG-0802): a multicentre, open-label, randomised, phase 3 study [J]. Lancet Oncol. 2011;12(8):735–42. 21783417 10.1016/S1470-2045(11)70184-X

[5] Reck M, Remon J, Hellmann MD. First-Line Immunotherapy for Non-Small-Cell Lung Cancer [J]. J Clin Oncol. 2022;40(6):586–97. 34985920 10.1200/JCO.21.01497

[6] Baek YH, Kang EJ, Hong S, et al. Survival outcomes of patients with nonsmall cell lung cancer concomitantly receiving proton pump inhibitors and immune checkpoint inhibitors [J]. Int J Cancer. 2022;150(8):1291–300. 34877670 10.1002/ijc.33892

[7] de Castro G, Jr., Kudaba I, Wu Y L, et al. Five-Year Outcomes With Pembrolizumab Versus Chemotherapy as First-Line Therapy in Patients With Non-Small-Cell Lung Cancer and Programmed Death Ligand-1 Tumor Proportion Score >/= 1% in the KEYNOTE-042 Study [J]. J Clin Oncol. 2023;41(11):1986–91. 36306479 10.1200/JCO.21.02885 PMC10082298

[8] Fukuoka M, Wu YL, Thongprasert S, et al. Biomarker analyses and final overall survival results from a phase III, randomized, open-label, first-line study of gefitinib versus carboplatin/paclitaxel in clinically selected patients with advanced non-small-cell lung cancer in Asia (IPASS) [J]. J Clin Oncol. 2011;29(21):2866–74. 21670455 10.1200/JCO.2010.33.4235

[9] Peng S, Wang R, Zhang X, et al. EGFR-TKI resistance promotes immune escape in lung cancer via increased PD-L1 expression [J]. Mol Cancer. 2019;18(1):165. 31747941 10.1186/s12943-019-1073-4 PMC6864970

[10] Wang Z, Zhang L, Xu W, et al. The Multi-Omics Analysis of Key Genes Regulating EGFR-TKI Resistance, Immune Infiltration, SCLC Transformation in EGFR-Mutant NSCLC [J]. J Inflamm Res. 2022;15:649–67. 35140497 10.2147/JIR.S341001 PMC8818984

[11] Wang J, Sanmamed M F, Datar I, et al. Fibrinogen-like Protein 1 Is a Major Immune Inhibitory Ligand of LAG-3 [J]. Cell. 2019;176(1–2):334–47e12. 30580966 10.1016/j.cell.2018.11.010 PMC6365968

[12] Tsai HI, Wu Y, Liu X, et al. Engineered small extracellular vesicles as a FGL1/PD-L1 dual-targeting delivery system for alleviating immune rejection [J]. Adv Sci (Weinh). 2022;9(3):e2102634. 34738731 10.1002/advs.202102634 PMC8787398

[13] Qian W, Zhao M, Wang R, et al. Fibrinogen-like protein 1 (FGL1): the next immune checkpoint target [J]. J Hematol Oncol. 2021;14(1):147. 34526102 10.1186/s13045-021-01161-8 PMC8444356

[14] Bie F, Wang G, Qu X, et al. Loss of FGL1 induces epithelial-mesenchymal transition and angiogenesis in LKB1 mutant lung adenocarcinoma [J]. Int J Oncol. 2019;55(3):697–707. 31322182 10.3892/ijo.2019.4838

[15] Zhang Y, Qiao HX, Zhou YT, et al. Fibrinogen-like-protein 1 promotes the invasion and metastasis of gastric cancer and is associated with poor prognosis [J]. Mol Med Rep. 2018;18(2):1465–72. 29845203 10.3892/mmr.2018.9097 PMC6072172

[16] Lv Z, Cui B, Huang X, et al. FGL1 as a novel mediator and biomarker of malignant progression in clear cell renal cell carcinoma [J]. Front Oncol. 2021;11:756843. 34956878 10.3389/fonc.2021.756843 PMC8695555

[17] Jin H, Kang GY, Jeon S, et al. Identification of molecular signatures involved in radiation-induced lung fibrosis [J]. J Mol Med (Berl). 2019;97(1):37–47. 30406363 10.1007/s00109-018-1715-9 PMC6326977

[18] Tang XY, Xiong YL, Zhao YB, et al. Dual immunological and proliferative regulation of immune checkpoint FGL1 in lung adenocarcinoma: The pivotal role of the YY1-FGL1-MYH9 axis [J]. Front Immunol. 2022;13:1014053. 36268014 10.3389/fimmu.2022.1014053 PMC9577086

[19] Yu HT, Yu M, Li CY, et al. Specific expression and regulation of hepassocin in the liver and down-regulation of the correlation of HNF1alpha with decreased levels of hepassocin in human hepatocellular carcinoma [J]. J Biol Chem. 2009;284(20):13335–47. 19304666 10.1074/jbc.M806393200 PMC2679433

[20] Yousef EH, El-Magd NFA, El Gayar AM. Norcantharidin potentiates sorafenib antitumor activity in hepatocellular carcinoma rat model through inhibiting IL-6/STAT3 pathway [J]. Transl Res. 2023;260:69–82. 37257560 10.1016/j.trsl.2023.05.005

[21] Byvoet P, Shepherd GR, Hardin JM, et al. The distribution and turnover of labeled methyl groups in histone fractions of cultured mammalian cells [J]. Arch Biochem Biophys. 1972;148(2):558–67. 5063076 10.1016/0003-9861(72)90174-9

[22] D. B, C. S, Allis D. The language of covalent histone modications. [J]. Nature. 2000, 403: 41–45. 10.1038/47412 10638745

[23] Fischle W, Franz H, Jacobs SA, et al. Specificity of the chromodomain Y chromosome family of chromodomains for lysine-methylated ARK(S/T) motifs [J]. J Biol Chem. 2008;283(28):19626–35. 18450745 10.1074/jbc.M802655200 PMC2443675

[24] Black JC, Van Rechem C, Whetstine JR. Histone lysine methylation dynamics: establishment, regulation, and biological impact [J]. Mol Cell. 2012;48(4):491–507. 23200123 10.1016/j.molcel.2012.11.006 PMC3861058

[25] Berry WL, Janknecht R. KDM4/JMJD2 histone demethylases: epigenetic regulators in cancer cells [J]. Cancer Res. 2013;73(10):2936–42. 23644528 10.1158/0008-5472.CAN-12-4300 PMC3655154

[26] Johnson DE, O’Keefe RA, Grandis JR. Targeting the IL-6/JAK/STAT3 signalling axis in cancer [J]. Nat Rev Clin Oncol. 2018;15(4):234–48. 29405201 10.1038/nrclinonc.2018.8 PMC5858971

[27] He F, Xiao H, Cai Y, et al. NSD1 promotes esophageal cancer tumorigenesis via HIF1alpha signaling [J]. Cell Biol Toxicol. 2023;39(4):1835–50. 36522543 10.1007/s10565-022-09786-2

[28] Liu M, Qin Y, Hu Q, et al. SETD8 potentiates constitutive ERK1/2 activation via epigenetically silencing DUSP10 expression in pancreatic cancer [J]. Cancer Lett. 2021;499:265–78. 33232789 10.1016/j.canlet.2020.11.023

[29] Kim H, Kim D, Choi SA, et al. KDM3A histone demethylase functions as an essential factor for activation of JAK2-STAT3 signaling pathway [J]. Proc Natl Acad Sci U S A. 2018;115(46):11766–71. 30377265 10.1073/pnas.1805662115 PMC6243239

[30] Zeng J, Zhang Y, Shang Y, et al. CancerSCEM: a database of single-cell expression map across various human cancers [J]. Nucleic Acids Res. 2022;50(D1):D1147–55. 34643725 10.1093/nar/gkab905 PMC8728207

[31] Mizuno H, Kitada K, Nakai K, et al. PrognoScan: a new database for meta-analysis of the prognostic value of genes [J]. BMC Med Genomics. 2009;2:18. 19393097 10.1186/1755-8794-2-18 PMC2689870

[32] Zhong P, Nakata K, Oyama K, et al. Blockade of histamine receptor H1 augments immune checkpoint therapy by enhancing MHC-I expression in pancreatic cancer cells [J]. J Exp Clin Cancer Res. 2024;43(1):138. 38715057 10.1186/s13046-024-03060-5 PMC11077718

[33] Li T, Fan J, Wang B, et al. TIMER: A Web Server for Comprehensive Analysis of Tumor-Infiltrating Immune Cells [J]. Cancer Res. 2017;77(21):e108–10. 29092952 10.1158/0008-5472.CAN-17-0307 PMC6042652

[34] Nelson JD, Denisenko O, Bomsztyk K. Protocol for the fast chromatin immunoprecipitation (ChIP) method [J]. Nat Protoc. 2006;1(1):179–85. 17406230 10.1038/nprot.2006.27

[35] Zheng Q, Dong H, Mo J, et al. A novel STAT3 inhibitor W2014-S regresses human non-small cell lung cancer xenografts and sensitizes EGFR-TKI acquired resistance [J]. Theranostics. 2021;11(2):824–40. 33391507 10.7150/thno.49600 PMC7738869

[36] Gyorffy B. Transcriptome-level Discovery of Survival-Associated Biomarkers and Therapy Targets in Non-Small-Cell Lung Cancer [J]. Br J Pharmacol, 2024;181(3):362–74. 10.1111/bph.16257 37783508

[37] Li H, Zhu YZ, Xu L, et al. Exploring new frontiers: cell surface vimentin as an emerging marker for circulating tumor cells and a promising therapeutic target in advanced gastric Cancer [J]. J Exp Clin Cancer Res. 2024;43(1):129. 38685125 10.1186/s13046-024-03043-6 PMC11059585

[38] Cheng KP, Ou HY, Hung HC, et al. Unsaturated fatty acids increase the expression of hepassocin through a signal transducer and activator of transcription 3-dependent pathway in HepG2 Cells [J]. Lipids. 2018;53(9):863–9. 30460699 10.1002/lipd.12099

[39] Wu Q, Young B, Wang Y, et al. Recent advances with KDM4 inhibitors and potential applications [J]. J Med Chem. 2022;65(14):9564–79. 35838529 10.1021/acs.jmedchem.2c00680 PMC9531573

[40] Alix-Panabières C. The future of liquid biopsy [J]. Nature, 2020;579(7800):S9. 10.1038/d41586-020-00844-5 32214259

[41] Zhan Q, Liu B, Situ X, et al. New insights into the correlations between circulating tumor cells and target organ metastasis [J]. Signal Transduct Target Ther. 2023;8(1):465. 38129401 10.1038/s41392-023-01725-9 PMC10739776

[42] Zeinali M, Lee M, Nadhan A, et al. High-Throughput Label-Free Isolation of Heterogeneous Circulating Tumor Cells and CTC Clusters from Non-Small-Cell Lung Cancer Patients [J]. Cancers (Basel). 2020;12(1):127. 10.3390/cancers12010127 PMC7016759 31947893

[43] Satelli A, Mitra A, Brownlee Z, et al. Epithelial-mesenchymal transitioned circulating tumor cells capture for detecting tumor progression [J]. Clin Cancer Res. 2015;21(4):899–906. 25516888 10.1158/1078-0432.CCR-14-0894 PMC4334736

[44] Xie X, Wang L, Wang X, et al. Evaluation of cell surface vimentin positive circulating tumor cells as a diagnostic biomarker for lung cancer [J]. Front Oncol. 2021;11:672687. 34055642 10.3389/fonc.2021.672687 PMC8162210

[45] Satelli A, Brownlee Z, Mitra A, et al. Circulating tumor cell enumeration with a combination of epithelial cell adhesion molecule- and cell-surface vimentin-based methods for monitoring breast cancer therapeutic response [J]. Clin Chem. 2015;61(1):259–66. 25336717 10.1373/clinchem.2014.228122 PMC4360893

