# Supplementary figures and images for: FGL1: a novel biomarker and target for non-small cell lung cancer, promoting tumor progression and metastasis through KDM4A/STAT3 transcription mechanism
Source: J Exp Clin Cancer Res. 2024 Aug 1;43:213. doi: 10.1186/s13046-024-03140-6 (PMC11293164; doi:10.1186/s13046-024-03140-6)

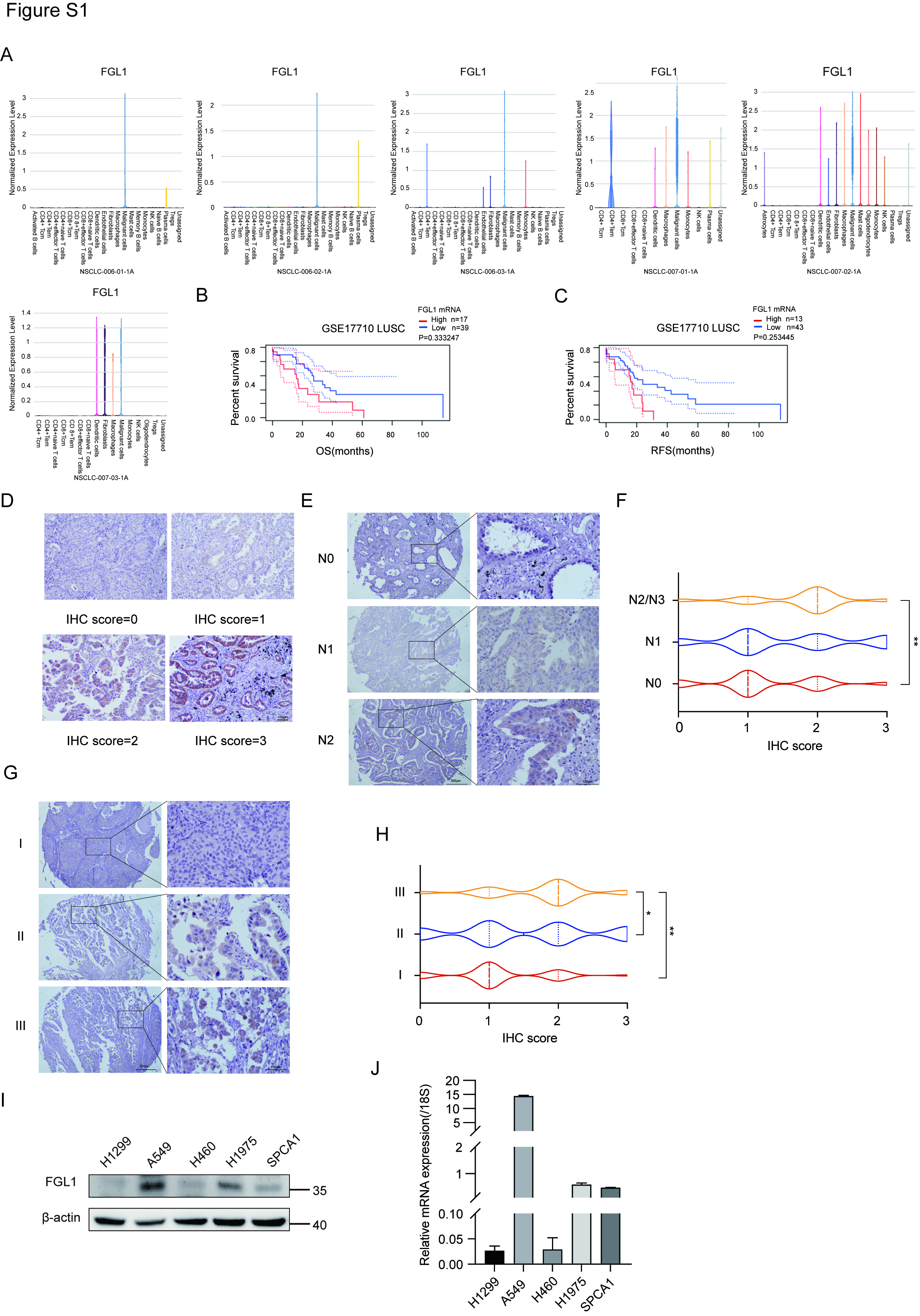

Supplement: Supplementary file 1 — Supplementary Material 1. Fig. S1. (A) Cell component comparison between single-cell samples in NSCLC using the CancerSCEM database. (B) Analysis of the correlation between FGL1 and OS using the Prognoscan database (GSE17710). (C) Analysis of the correlation between FGL1 and RFS using the Prognoscan database (GSE17710). (D) Scoring based on the intensity of FGL1 expression: 0 points for "negative", 1 point for "weak positive", 2 points for "positive", and 3 points for "strong positive". (E) Representative images of tissue slices from patients with lymph node stages of N0, N1, and N2. (F) According to lymph node staging, the expression of FGL1 was analyzed in 98 patients with cancer. **P < 0.01. (G) Representative images of tissue slices from patients with clinical stages I, II, and III. (H) According to the clinical staging, the expression of FGL1 was analyzed in 98 patients with cancer. *P < 0.05, **P < 0.01. (I) The expression of FGL1 protein was measured on five NSCLC cancer cell lines (A549, H1299, H1975, H460, and SPCA1). (J) The expression of FGL1 mRNA was measured on five NSCLC cancer cell lines (A549, H1299, H1975, H460, and SPCA1). [file 13046_2024_3140_MOESM1_ESM.tif]

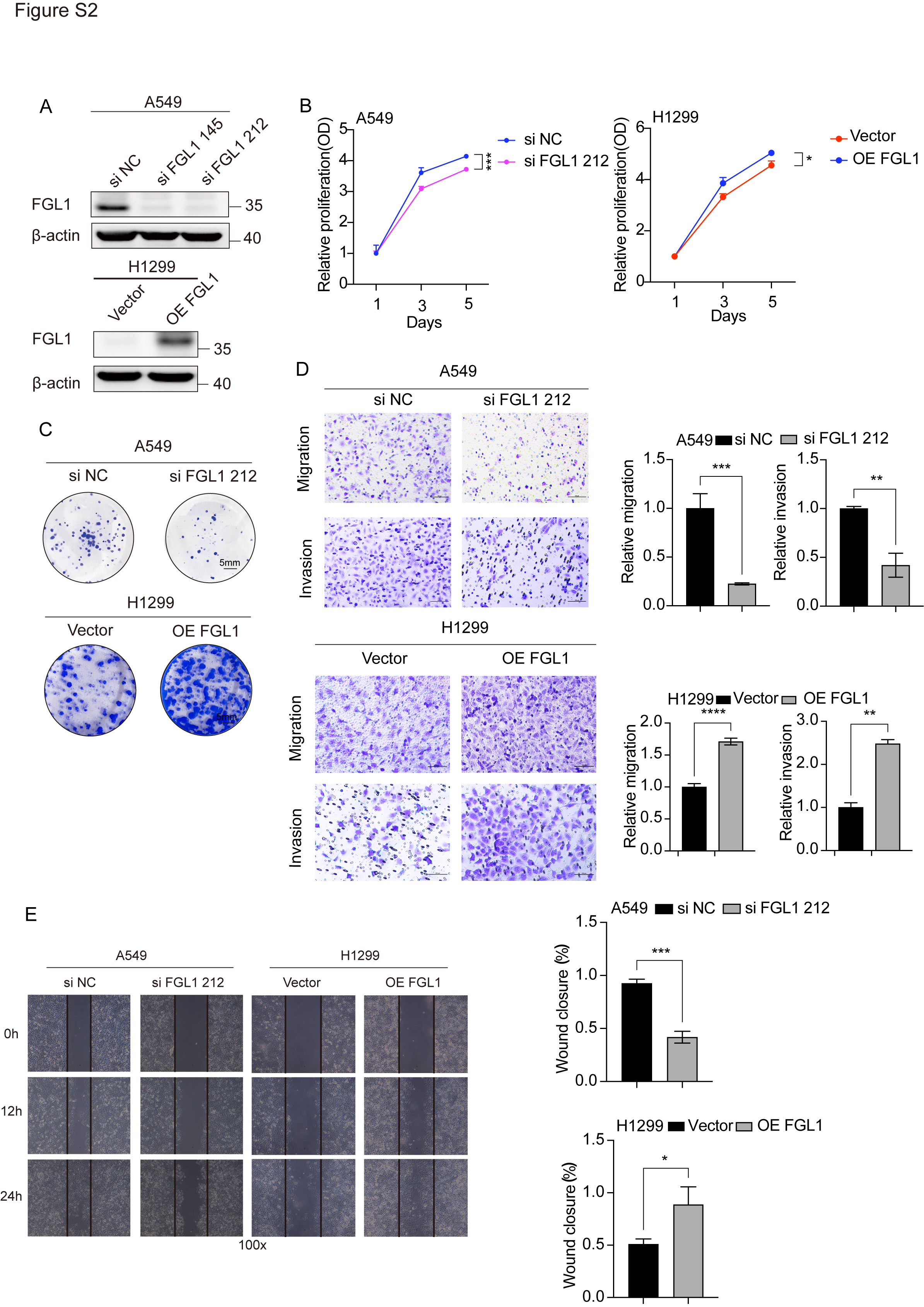

Supplement: Supplementary file 2 — Supplementary Material 2. Fig. S2. (A) Validation of FGL1 protein expression after FGL1 knockdown using siRNA in A549 cells and FGL1 overexpression in H1299 cells. (B) MTS assay to assess the effect of FGL1 knockdown on the proliferation of A549 cells and FGL1 overexpression on the proliferation of H1299 cells. (C) Clonogenic assay for evaluating the effect of FGL1 knockdown on the proliferation of A549 cells and FGL1 overexpression on the proliferation of H1299 cells. Scale bars were 5 mm. (D) Transwell assay to determine the effect of FGL1 knockdown on the migration and invasion of A549 cells and FGL1 overexpression on the migration and invasion of H1299 cells. Scale bars were 100 μm. (E) Scratch assay to measure the effect of FGL1 knockdown on the migration of A549 cells and FGL1 overexpression on the migration of H1299 cells. The images are magnified by 100 times. Student’s t-test was used for analysis and error bars represent mean ± SD. *P < 0.05, **P < 0.01, ***P < 0.001, ****P < 0.0001. [file 13046_2024_3140_MOESM2_ESM.tif]

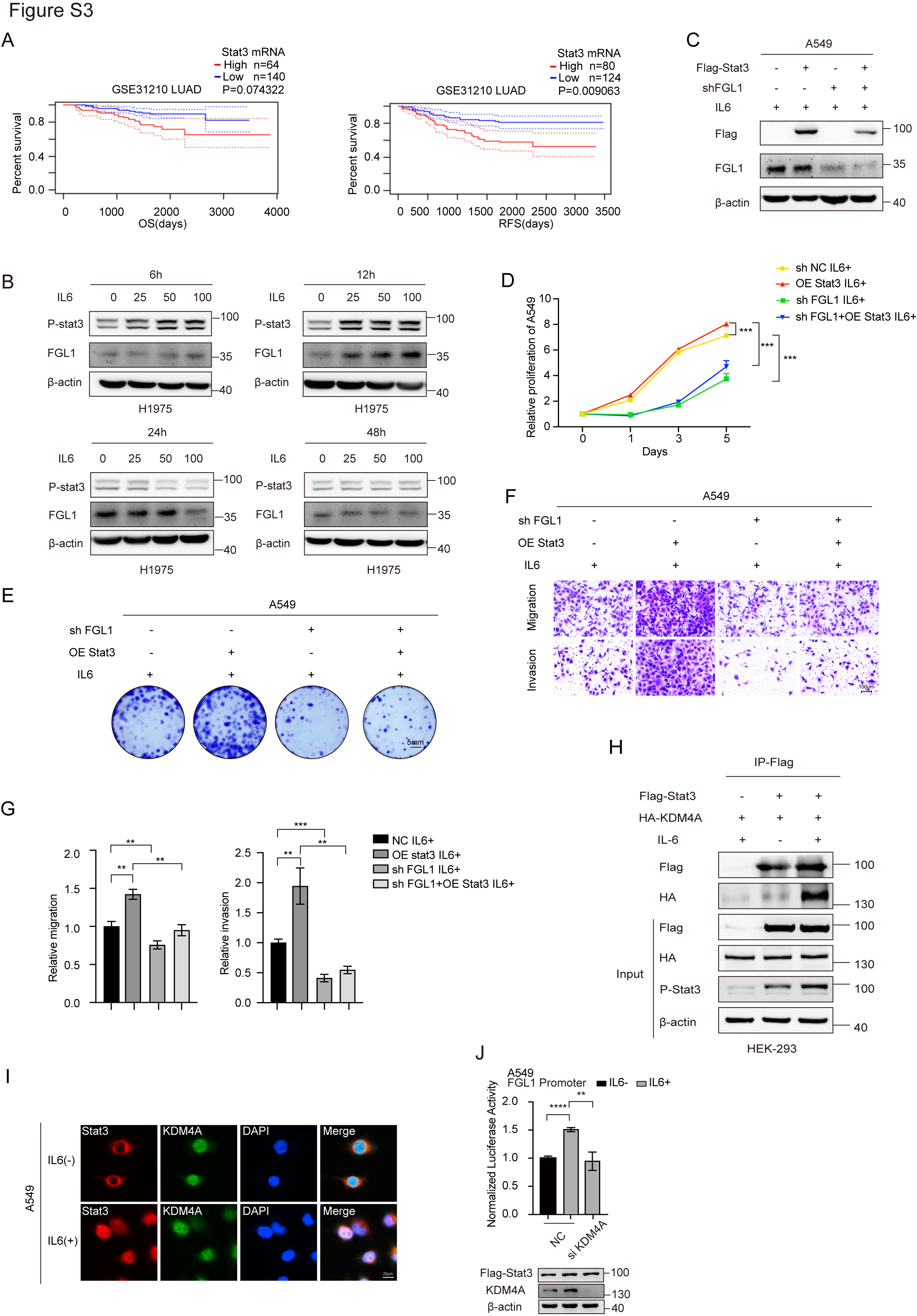

Supplement: Supplementary file 3 — Supplementary Material 3. Fig. S3. (A) Analysis of the GSE31210 dataset to assess the effect of Stat3 expression on OS and RFS in patients with LUAD. (B) The effect of IL6 stimulation at different time points and concentrations on Stat3 phosphorylation and FGL1 protein expression. (C) Validation of Flag-Stat3 and FGL1 protein expression in cells under different treatment conditions. (D) MTS assay to assess the effect of FGL1 knockdown and Stat3 overexpression on cell proliferation. Scale bars were 100 μm. (E) Colony formation assay to validate the effect of FGL1 knockdown and Stat3 overexpression on cell proliferation. Scale bars were 5 mm. (F) Transwell assay to determine the effect of FGL1 knockdown and Stat3 overexpression on cell migration and invasion. Scale bars were 100 μm. (G) Statistical analysis of Figure D. Student’s t-test was used for analysis and error bars represent mean ± SD. **P < 0.01, ***P < 0.001. (H) Co-IP experiments showing the interaction between exogenous Stat3 and KDM4A in HEK-293 cells. (I) The distribution of KDM4A and Stat3 with or without IL6 in immunofluorescence staining of A549 cells. Cells were stained with anti-DAPI (Blue), anti-Stat3 (Red), and anti-KDM4A (Green). Scale bars are 20 μm. (J) Luciferase assay demonstrating the effect of KDM4A on FGL1 promoter activity. Student’s t-test was used for analysis and error bars represent mean ± SD. **P < 0.01, ***P < 0.001, ****P < 0.0001. Scale bars were 100 μm. [file 13046_2024_3140_MOESM3_ESM.tif]

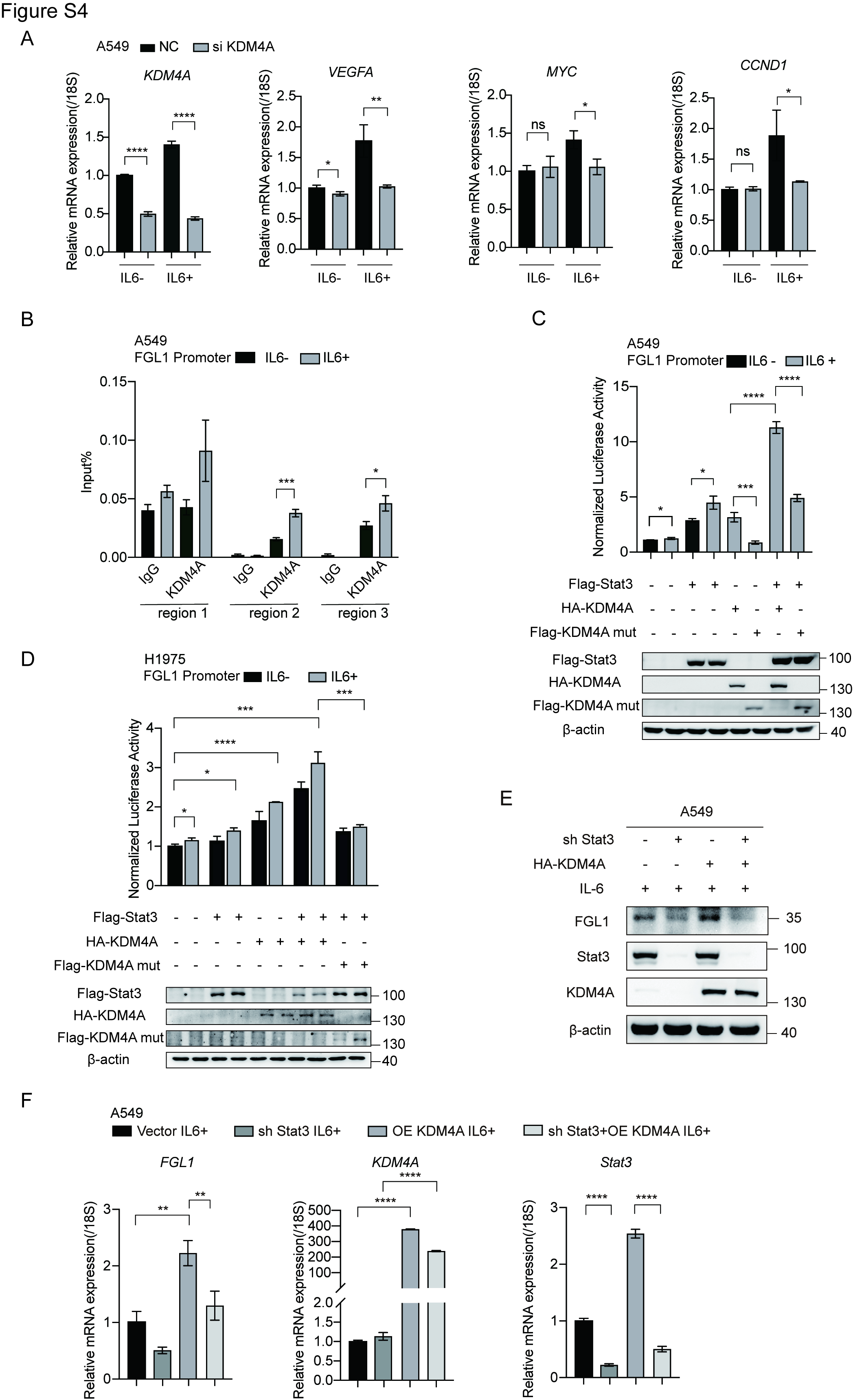

Supplement: Supplementary file 4 — Supplementary Material 4. Fig. S4. (A) Effect of KDM4A knockdown on the mRNA expression of classical target genes of Stat3. (B) Recruitment of KDM4A to Stat3 binding regions on the FGL1 promoter. (C-D) Changes in FGL1 promoter activity under different treatment conditions in A549 (C) and H1975 cells (D). (E–F) Changes in FGL1 protein (E) and mRNA (F) expression upon stable Stat3 knockdown and simultaneous overexpression of KDM4A in A549 cells. Student’s t-test was used for analysis, and error bars represent mean ± SD. *P < 0.05, **P < 0.01, ***P < 0.001, ****P < 0.0001, and ns stands for no significance. [file 13046_2024_3140_MOESM4_ESM.tif]

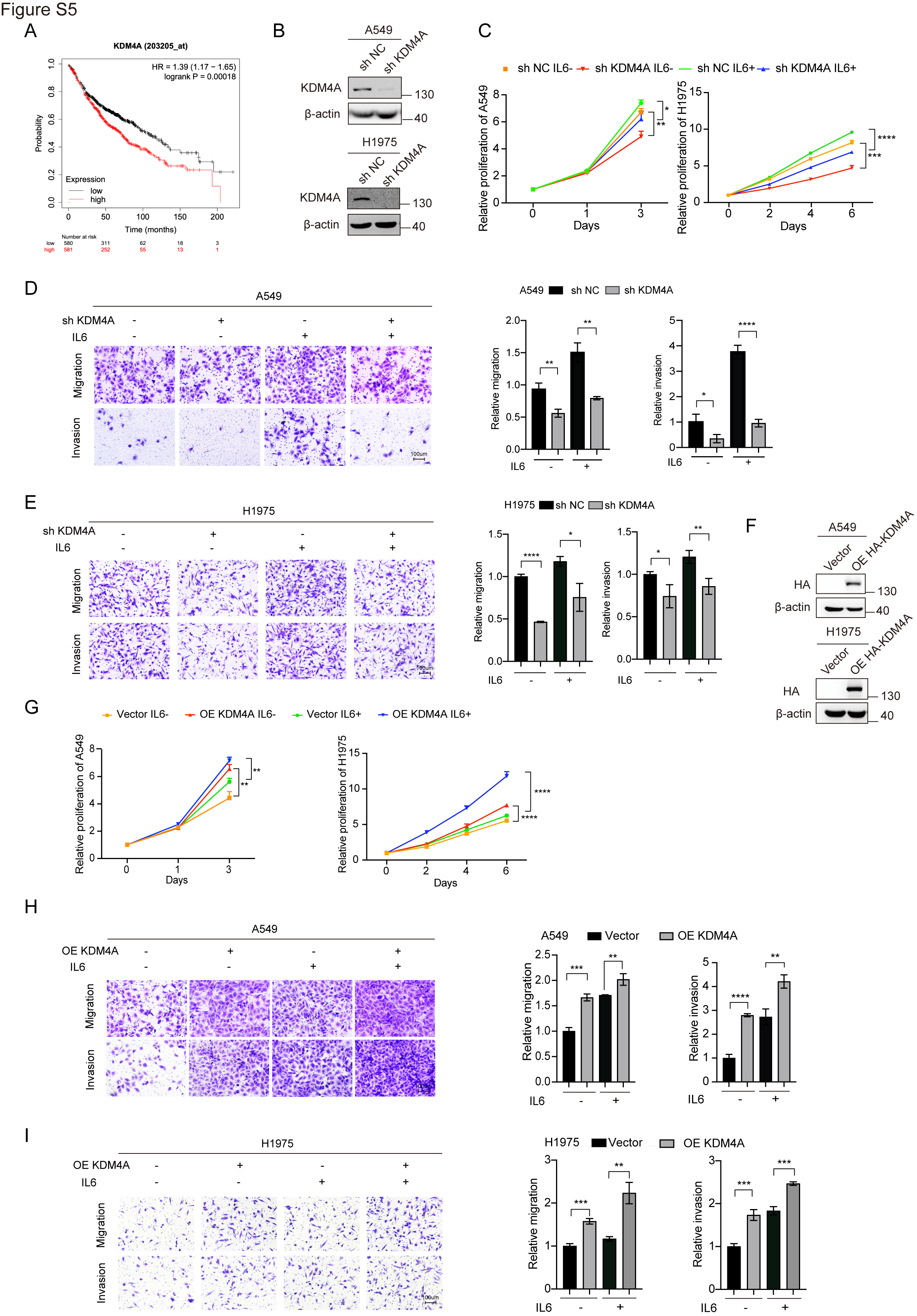

Supplement: Supplementary file 5 — Supplementary Material 5. Fig. S5. (A) Analysis of KDM4A expression and its effect on OS in LUAD patients using the KM plotter. (B) Validation of KDM4A protein expression under different treatment conditions in A549 and H1975 cells. (C) MTS assay exhibiting the effect of KDM4A knockdown on A549 and H1975 cell proliferation. (D-E) Transwell assay showing the effect of KDM4A knockdown on the migration and invasion of A549 and H1975 cells. Scale bars were 100 μm. (F) Validation of KDM4A protein expression under different treatment conditions in A549 and H1975 cells. (G) MTS assay exhibiting the effect of KDM4A overexpression on the proliferation of A549 and H1975 cells. (H) Transwell assay illustrating the effect of KDM4A overexpression on the migration and invasion of A549 cells. Scale bars were 100 μm. (I) Transwell assay displaying the effect of KDM4A overexpression on the migration and invasion of H1975 cells. Student’s t-test was used for analysis, and error bars represent mean ± SD. *P < 0.05, **P < 0.01, ***P < 0.001, ****P < 0.0001. Scale bars were 100 μm. [file 13046_2024_3140_MOESM5_ESM.tif]

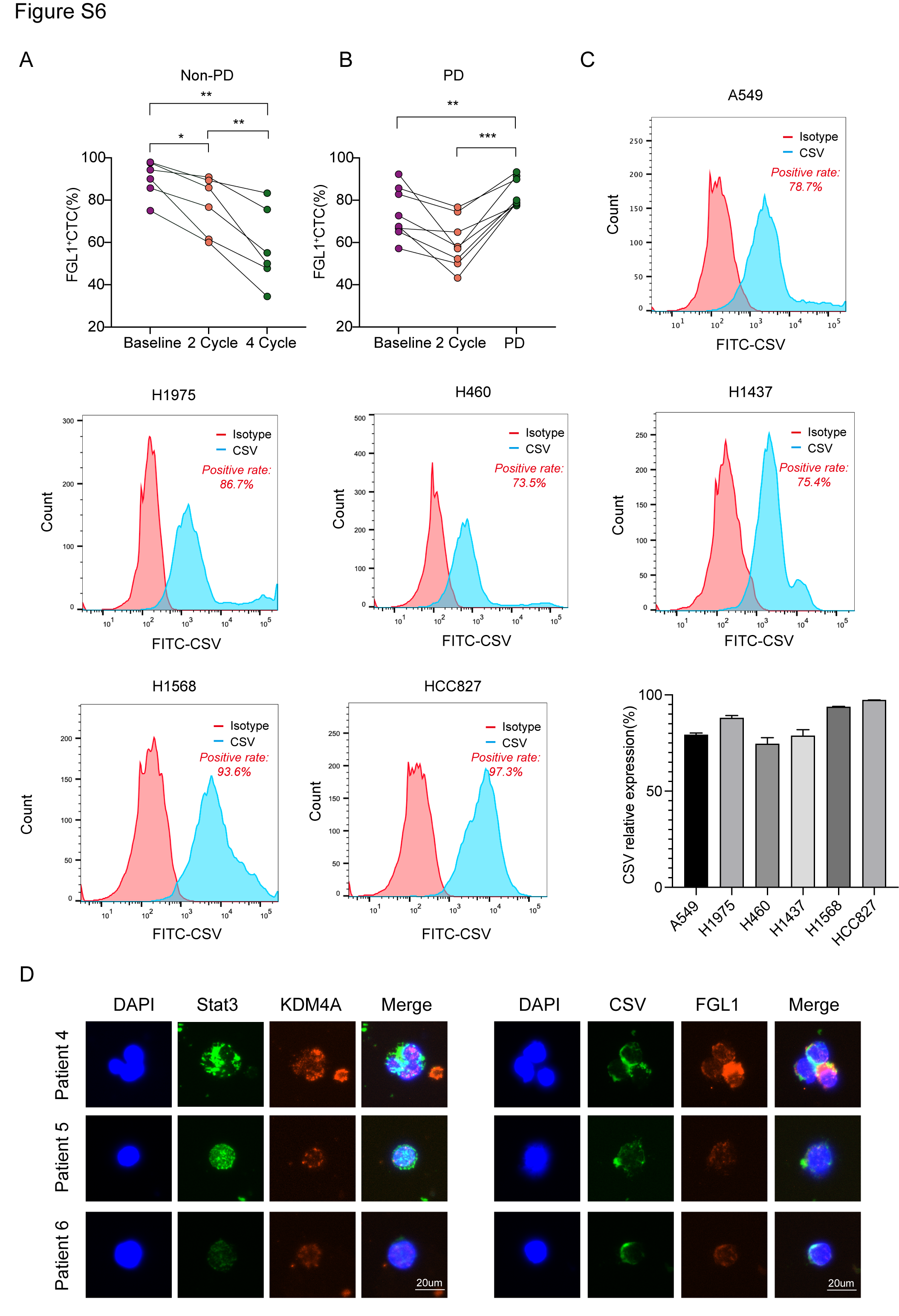

Supplement: Supplementary file 6 — Supplementary Material 6. Fig. S6. (A) Changes in FGL1 expression on CTCs from patients who showed a non-PD response after 2 and 4 cycles of evaluation. Student’s t-test was used for analysis, and error bars represent mean ± SD. *P < 0.05, **P < 0.01, ***P < 0.001. (B) Changes in FGL1 expression on CTCs from patients who showed PD at the final assessment. (C) Bar graphs showing the expression levels of CSV in NSCLC cell lines. (D) The distribution of KDM4A、Stat3、CSV and FGL1 by immunofluorescence staining in CTCs. Cells were stained with anti-DAPI (Blue), anti-Stat3 and anti-CSV(Green), anti-KDM4A and anti-FGL1 (Red). Scale bars were 20 μm. [file 13046_2024_3140_MOESM6_ESM.tif]
