# Supplementary material for: FGL1: a novel biomarker and target for non-small cell lung cancer, promoting tumor progression and metastasis through KDM4A/STAT3 transcription mechanism
Source: J Exp Clin Cancer Res. 2024 Aug 1;43:213. doi: 10.1186/s13046-024-03140-6 (PMC11293164; doi:10.1186/s13046-024-03140-6)
Supplement: Supplementary file 7 — Supplementary Material 7. [file 13046_2024_3140_MOESM7_ESM.docx]

**Materials and methods**

**Plasmids and antibodies**

FGL1 and Stat3 expression plasmids and FGL1 promoter reporter plasmid were purchased from Shanghai GeneChem Company. Plasmids of KDM4A were constructed in our laboratory.

The antibodies used in this study were: anti-FLAG (cat#PA1-984B，Sigma)，anti-HA（cat#51064-2-AP Proteintech),anti-β-actin (cat#60008-1-Ig, Proteintech) ,anti-FGL1 (cat#ab275091,Abcam),anti-Stat3 (cat#9139,Cell Signaling Technology),anti-pStat3 (cat#9145,Cell Signaling Technology),anti-KDM4A,(cat#5328,Cell Signaling Technology),anti-H3K9me3(cat#13969,Cell Signaling Technology),anti-IgG(cat#A7016、cat#A7028，Beyotime Biotechnology),anti-CSV(cat#H00007431-M08,Abnova).

**siRNA and lentivirus**

siRNA duplexes against FGL1、Stat3、KDM4A were purchased from Jintuosi(Wuhan) Biotechnology. Sequence of siFGL1:1#5′GCAGGGAAAUUUCGGCGCUTT3′ 2#5′ GCUUGAGACCCGGGUCAAATT3′; siStat3:5′ GGAAACAACCAGUCAGUGATT 3′ siKDM4A:5′ GUGAUGAUGAGACAUCUGADTDT3′; For lentivirus-delivered RNAi, lentiviral productions of FGL1 and Stat3 were purchased from Shanghai GeneChem Company. Lentiviral production of FGL1 targeted the same sequence as siFGL1#2. Lentiviral production of Stat3 targeted the same sequence as siStat3.Lentiviral productions of KDM4A were purchased from Jintuosi (Wuhan) Biotechnology. Target sequence for KDM4A lentivirus: GTCAAATTTGAAGAGCTTA

**Patient eligibility and recruitment**

A total of 65 patients with locally advanced or metastatic non-small cell lung cancer (NSCLC) receiving first-line immunotherapy at the Department of Medical Oncology II, The First Hospital of China Medical University, were recruited for this study. Peripheral blood samples were collected from these patients. Clinical information of the patients was recorded, and all patients underwent efficacy evaluation at least every 6-8 weeks after receiving immunotherapy. Treatment response was assessed according to the Response Evaluation Criteria in Solid Tumors (RECIST 1.1) and categorized as complete response (CR), partial response (PR), stable disease (SD), or progressive disease (PD). This study was approved by the Ethics Committee of The First Hospital of China Medical University (Protocol:AF-SOP-07-1,1-01), and all enrolled patients provided informed consent. The study methodology adhered to the principles outlined in the Helsinki Declaration.

**Peripheral Blood Collection and Processing**

Upon admission, fasting peripheral blood samples of 10 ml were collected from the patients via venipuncture. Within 2 hours of collection, peripheral blood mononuclear cells (PBMCs) were isolated to ensure optimal enrichment of CTCs. After isolation, the PBMCs were placed in a mixture solution containing 900 µl of high-quality fetal bovine serum and 100 µl of DMSO. The mixture was then stored in a cryovial and placed in a freezing box containing isopropanol. The freezing box was promptly transferred to a -80°C freezer for storage.

**Enrichment of CTCs^[44]^**

(1) Negative enrichment preheat a water bath to 56°C. Thaw the cells from the cryovial within 1 minute. Place the thawed cells in 3 ml of Easy Buffer and centrifuge to extract PBMCs for negative enrichment. Deplete the CD45+ cell population from PBMCs using the EasySep Human CD45 kit (StemCell Technologies).

(2) Positive enrichment use 84-1 (CSV-specific labeling antibody) to sort the CD45- cell population and obtain the CSV+ cell subset. Followed by binding with Anti-mouse IgG microbeads (Miltenyi Biotec, Bergisch Gladbach, Germany). Then, use magnetic columns (Miltenyi Biotec) to enrich the cells labeled with 84-1 antibody into a 15 ml centrifuge tube, resulting in CSV+CD45- cells for further research.

**Immunofluorescence Technique Resuspend**

The isolated cells obtained from the above step using MACS Buffer and incubate with 84-1 antibody in a 37°C water bath for 1 hour. After incubation, evenly distribute 100 µl of the cell suspension onto a glass slide using a Cytospin 4. Fix the cells on the glass slide with 4% paraformaldehyde for 10 minutes. Permeabilize the cells with 1% Triton X-100. Then, incubate with the primary antibody against FGL1 and store overnight at 4°C.

On the following day, incubate the cells with AF-488 and AF-555 secondary antibodies for 1 hour to detect FGL1^+^CSV^+^ CTCs. After 1 hour, stain the cell nuclei with DAPI. After completing the experiment, count the cells using a fluorescence microscope (Zeiss).

**Cell culture and transfections**

The A549、NCI-H1975、NCI-H1299、H460、SPCA1、BEAS-2B cells were routinely cultured in RMPI-1640 medium (Gibco).The HEK-293 cells were cultured in Dulbercco’s modified Eagle’s medium (DMEM, GIBCO).All culture media were supplemented with 10% fetal bovine serum and cells were maintained at 37°C in a humidified atmosphere with 5% CO2.All cells were cultured in serum-free DMEM or RMPI-1640 for 12 hours prior to stimulation with IL6.

Transfections of plasmids and siRNAs were performed according to manufacturer’s instructions of jetPRIME transfection reagents (Polyplus-transfection, Cat#101000046).

**Luciferase assay**

Cell lines were cotransfected with the listed constructs according to the manufacturer’s instructions. After 24 hours of transfection, cells were harvested using 80 μL of passive lysis buffer (Promega). Luciferase activities were analyzed using a Promega dual-luciferase reporter assay system. Firefly luciferase activity was normalized to the activity of Renilla luciferase control. Relative luciferase activity was analyzed using the luminometer Lumat LB 9507(Berthold Technologies, Germany).

**RNA isolation and quantitative real-time PCR assays**

Total RNA was extracted using Trizol reagent (Vazyme, Cat#R401-01) and cDNA wrer reversed by PrimeScript™ RT-PCR Kit (TAKARA,cat#RR036A) .Real-time qPCR assays were performed using the ChamQ Universal SYBR qPCR Master Mix（Vazyme #Q711）

according to the manuscription’s instructions on LightCycler96 (Roche).Sequences of primers used to detect mRNA expression were listed as follows:FGL1:5′-3′：ATGGCAAAGGTGTTCAGTTTCA 3′-5′:ACAATCTGCATACTGCCTCTTGStat3:5′-3′:TCCTGAAGCTGACCCAGGTA 3′-5′:TATTGCTGCAGGTCGTTGGT；KDM4A：5′-3′:TGTTCTCGGTGTTCAGCCAATGC 3′-5′:ACCTTGCTTCCAGAATTGCCACAG;18s:5′-3′:TTGACGGAAGGGCACCACCAG 3′-5′:GCACCACCACCCACGGAATCG

**Immunoprecipitation and western blotting**

In the Co-immunoprecipitation (Co-IP) experiment, whole cell lysates were extracted and subjected to equal protein treatment. Immunoprecipitation was performed using antibodies targeting Stat3, KDM4A, HA, or the anti-FLAG M2 resin (Sigma). Western blotting analysis was conducted on the crude extracts and immunocomplexes. Western blotting assays were performed by the standard process introduced in our previous study.

**Immunofluorescence**

The cells were fixed in a 4% paraformaldehyde solution for 15 minutes, permeabilized with Triton X-100 for 10 minutes, and then blocked with 1% donkey serum albumin at room temperature for 1 hour. The cells were incubated with primary antibodies overnight at 4°C, subsequently conjugated secondary antibody(Invitrogen cat#A21202、cat#A10040). Nuclei were stained with DAPI (Beyotime cat#C1006).

**ChIP assay^[45]^**

Using standard protocols from Nature Protocols we performed ChIP, the cells were fixed with 1% formaldehyde (final concentration) for cross-linking. Next, the cells were harvested in lysis buffer and subjected to sonication on ice. IP of the chromatin solution was performed using either anti-Stat3, anti-KDM4A, or histone modification antibodies. The IP reaction was conducted by incubating the chromatin solution with protein A-Sepharose beads (Millipore cat#16-157) overnight at 4°C. Following IP, the protein-DNA complexes were washed sequentially with low salt buffer, high salt buffer, LiCl buffer, and TE buffer. The protein-DNA complexes were then eluted, reverse-crosslinked, and purified. Finally, the DNA was dissolved in TE buffer. After experiments, DNAs were used as templates for qPCR, and the primers were listed as follows：region1:5′-3′：GGGACCACAGGACAAATGGC 3′-5′:GTGTAATAGGCACTTGTTAGCTCC;region2:5′-3′：TCTGTGTGTGTGCAAATCACTT 3′-5′:TGAGCACTGACGATGTTTGGT;region3:5′-3′：CGTCAGTGCTCAAGTCCAGTA 3′-5′:TTTGCCATTTGTCCTGTGGTC

**Cell proliferation、colony formation and transwell assay**

All NSCLC cells were incubated for given periods and then harvest with trypan blue to count with cell counting plate. Adjust the same number of cells per well. To generate a cell proliferation curve, cells were evenly seeded into a 96-well plate. At specific time points, cell viability was measured using MTS assay with absorbance recorded at 490 nm. For the colony formation experiment, cells were cultured in a designated medium for a specified period and then fixed with 4% paraformaldehyde for subsequent staining coomassie blue dye. In the Transwell experiment, when cells were observed to have migrated through the membrane, they were fixed with 95% ethanol and stained with 0.1% crystal violet. Images were captured under a microscope.

**Immunohistochemical (IHC) analysis**

Tissue samples were fixed with 10% neutral formalin, embedded in paraffin, and sectioned into 4 μm thick slices. The samples underwent deparaffinization and hydration, followed by a 15-minute incubation in 3% H2O2 at room temperature. Subsequently, the samples were incubated overnight at 4℃ with anti-FGL1. Afterward, the slides were incubated with biotinylated goat anti-rabbit antibody for 1 hour, followed by staining with 3,3’-diaminobenzidine (DAB; Maixin Biotechnology, Fuzhou, China). Finally, counterstaining was performed using hematoxylin (Maixin Biotechnology).

IHC staining was performed on an lung Adenocarcinoma tissue microarray (Shanghai Outdo Biotech Company cat No.HLugA180Su11). Blinded evaluation of stained slides was performed, without prior knowledge of clinical information. *Based on tissue microarray IHC staining of FGL1, patients with “positive” and “strong positive” staining were assigned to the high-expression group, while those with “negative” or “weak positive” staining were assigned to the low-expression group^[32]^.*

**Animal experiments**

The whole mouse experiments were performed at the Institutional Animal Care and Use Committee of China Medical University.

A549 cells were respectively stably expressed sh NC and sh FGL1 lentivirus. All cells were suspended in 50ul culture medium and 50ul Matrigel (BD biosciences) and injected the number of 1×10^7^ into 4-week-old male BALB/c nude mice (Beijing HFK Bio-Technology). we monitored the mice every seven days for about 4 weeks which time the mice were killed in keeping with the policy of humane treatment.

*LLC cells were suspended in 50ul PBS and injected the number of 5×10^6^ into 4-week-old male BALB/c nude mice (Beijing HFK Bio-Technology). The FGL1 antibody and (or) KDM4A inhibitor (QC6352, MCE HY-104048) and FGL1 mAb (Bioxcell Cat#BE0332) were administered intraperitoneally to the mice at the scheduled intervals. QC6352 was administered once daily at a dose of 10mg/kg via intraperitoneal injection, while FGL1mAb was administered once every four days at a dose of 100ug per injection via intraperitoneal injection.* *We monitored the mice every four days for about 4 weeks which time the mice were killed in keeping with the policy of humane treatment.*

**Statistical analysis**

Graphpad prism 9.1.1 software was used for statistical analysis, statistical data were expressed as mean ± standard deviation (SD). Student’s t-test was used to determinate the significant difference. P ≤0.05 was considered statistically significant (**P*<0.05; ***P*<0.01; ****P*<0.001; *****P*<0.0001)

**Clinical and treatment information for 3 cases of NSCLC patients.**

**Patient 1**

The patient with a 20-year history of smoking, received a diagnosis of lung adenocarcinoma through bronchoscopy biopsy on February 28, 2023, classified as clinical stage T2aN3M1b, stage IV, indicating tumor-lymph node and bone metastasis. Next generation sequencing based genomic detection was performed and the results showed that the patient harbored the mutations of KRAS exon2 and TP53 exon5. PD-L1 testing was not performed due to insufficient tissue. Considering these findings, anti-PD-1 therapy and platinum-based chemotherapy included tislelizumab (100 mg), pemetrexed (0.8 g), and carboplatin (450 mg) every three weeks for four cycles. Clinical evaluation of the tumor based on response evaluation criteria in solid tumors RECIST version 1.1 showed partially response (PR) after 2 cycles, and a partial response was maintained after 4 cycles. Moreover, this patient has been treated with pemetrexed combined with immunotherapy so far. Treatment was ongoing as of the data cutoff date. Moreover, our findings revealed a continuous decrease in the patient's FGL1+CTC% from 90 at baseline, suggesting a correlation between the decrease in the proportion of FGL1+CTC% and the therapeutic efficacy in the patient.

**Patient 2**

The patient was a former smoker, diagnosed with stage IIIC (cT3N3M0) squamous cell carcinoma with a KRAS exon 2 mutation, exhibited PD-L1 at baseline (TPS=5%). The patient underwent first-line treatment with 2-cycle immunotherapy, comprising a TP regimen of Tislelizumab (100mg), albumin-bound paclitaxel(300mg), and carboplatin(400mg). After two cycles, the disease was progression (PD). It is noteworthy that a positive correlation exists between the treatment effect and the changes in FGL1+CTC%, suggesting that FGL1+CTC% may serve as a potential indicator of immune resistance.

**Patient 3**

The patient was a former smoker, received a diagnosis of lung adenocarcinoma through lung biopsy on October 22, 2022, classified as clinical stage T4N3M1c, stage IV, indicating tumor-lymph node and bone and Liver metastasis. Next-generation sequencing (NGS) showed no Class I mutations, exhibited PD-L1 at baseline (TPS=5%). The patient underwent first-line treatment with 2-cycle immunotherapy, comprising a TP regimen of Sintilimab (100mg), pemetrexed (0.8g), and carboplatin(400mg). After 6 cycles of treatment, the patient stopped anti-tumor therapy due to the development of immune-related ulcerative colitis. However, on June 05, 2023, the patient's lung CT examination unfortunately showed disease progression (PD). Therefore, our study concluded that the treatment effect is strongly associated with the alterations in FGL1+CTC%.
